# A Binary Supramolecular Assembly with Intense Fluorescence Emission, High pH Stability, and Cation Selectivity: Supramolecular Assembly-Induced Emission Materials

**DOI:** 10.34133/2019/1454562

**Published:** 2019-07-24

**Authors:** Xu Wang, Xin-Yue Lou, Xiao-Yu Jin, Feng Liang, Ying-Wei Yang

**Affiliations:** ^1^State Key Laboratory of Inorganic Synthesis and Preparative Chemistry, International Joint Research Laboratory of Nano-Micro Architecture Chemistry, College of Chemistry, Jilin University, 2699 Qianjin Street, Changchun 130012, China; ^2^The State Key Laboratory of Refractories and Metallurgy, School of Chemistry & Chemical Engineering, Wuhan University of Science and Technology, Wuhan 430081, China

## Abstract

We construct a fluorescent supramolecular system (TPE-Q_4_*⊂* DSP5) of excellent tolerance to a wide range of pH by the facile self-assembly of a new pillar[5]arene bearing disulfonated arms (DSP5) with an AIE-active tetraphenylethene-based tetratopic guest bearing four quaternary ammonium binding sites (TPE-Q_4_), which exhibits strong blue emission even in dilute aqueous solutions along with much higher quantum yield and longer fluorescence lifetime than TPE-Q_4_ itself. This appreciable property can be attributed to the supramolecular assembly-induced emission (SAIE) mechanism endowed by the host-guest inclusion complexation based on synthetic macrocycles. Remarkably, the enhanced fluorescence of the supramolecular assembly is quenched efficiently and exclusively by ferric ions in water with a high Stern–Volmer formula constant of 1.3 × 10^5^  mol^−1^, demonstrating the excellent cation selectivity and visualized responsiveness in ion sensing and detection.

## 1. Introduction

Fluorescent supramolecular assemblies represent an extraordinarily attractive class of functional systems, due to their integrated functionalities, highly tunable fluorescence properties, and good responsiveness towards external stimuli [1]. The inherent features of their fluorescent entities that can be further mediated by the rational design of supramolecular building blocks endow them extensive potentials in smart materials and light-emitting devices [2]. In 2001, Tang and coworkers discovered some propeller-like molecules with *π*-conjugated structures that exhibited weak fluorescence in solution but bright emission in aggregated states in sharp contrast to traditional fluorescent molecules of aggregation caused quenching (ACQ) and creatively named this phenomenon aggregation-induced emission (AIE) [3]. Many fluorophores with AIE properties (AIEgens) have been discovered and extensively investigated for applications in chemistry and materials science [4, 5]. The mechanism of fluorescent emission enhancement of AIEgens in the aggregated states mainly lies in the restriction of intramolecular motion (RIM) and the blockade of nonradiation energy dissipation channel from the excited state to ground state [4, 5].

Inspired by the mechanism of AIE, researchers have successfully developed a supramolecular pathway, namely, supramolecular assembly-induced emission (SAIE), to achieve effective emission enhancement of AIEgens in the ordered assembly states no matter in dilute solutions or aggregated solid states [2, 6–19]. In 2014, Tang and coworkers covalently linked a cyclodextrin with tetraphenylethene (TPE) and utilized self-inclusion complexation for RIM to achieve enhanced emission in aqueous solutions, which represented the prototype SAIE study and paved the way of recruiting supramolecular macrocycles to manipulate the fluorescent properties of AIEgens [6]. Considerable effort has been made in the rapid development of SAIE recently, accompanied by the synthesis of functional supramolecular assemblies with tunable emission [7–19]. Various types of supramolecular fluorescent assemblies based on AIEgens and functional synthetic macrocycles have been reported for applications in cell imaging, [11] fluorescent supramolecular polymers and gels [7–10, 13, 14], room-temperature phosphorescence [15], controlled drug delivery [16], and other stimuli-responsive materials [17–19]. But nonetheless, challenges in their preparation and application remain.

On the other hand, pillarenes, as an important class of macrocyclic hosts in the realm of supramolecular chemistry and advanced functional materials, are attracting more and more attention due to the unique pillar-like rigid structure, electron-rich cavity, well-studied host-guest properties, and facile modification methodology [20–22]. Functionalized pillarenes have played important roles in ion detection [23], antibacterial therapeutics [24], supramolecular nanovalves [25–29], and so on [30]. Significantly, by elaborate functionalization, pillarenes have been utilized as an important candidate of synthetic macrocycles in the construction of stimuli-responsive fluorescent materials through the marriage with AIEgens [2].

**Scheme 1:**
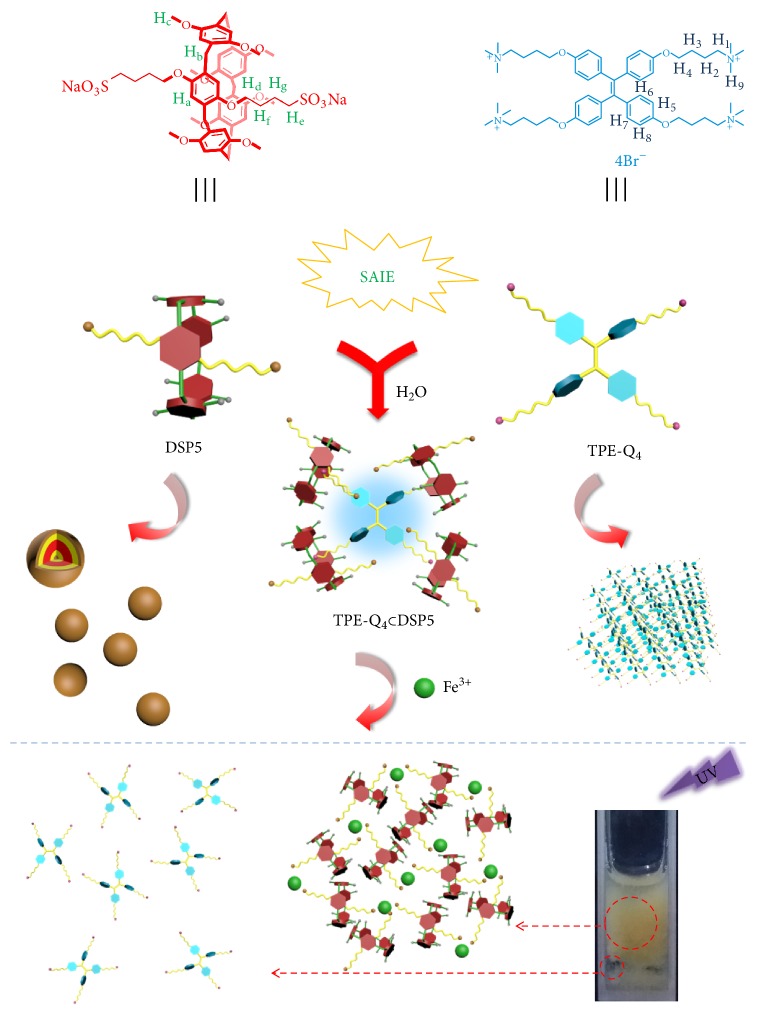
Chemical structures and cartoons of DSP5 and TPE-Q_4_ and the schematic presentation of their self-assembly into a fluorescent supramolecular system for the selective detection of Fe^3+^ ions.

Herein, we report on the fabrication of a pH-tolerant fluorescent system for specific cation recognition based on supramolecular self-assembly of disulfonated pillar[5]arene (DSP5) and a water-soluble TPE-based tetratopic guest (TPE-Q_4_) with four quaternary ammonium binding sites (Scheme 1). Because of the high binding affinity of DSP5 towards TPE-Q_4_, a supramolecular assembly (TPE-Q_4_*⊂* DSP5) was fabricated in aqueous solution via host-guest interaction, exhibiting bright blue emission in a wide range of pH conditions. Meanwhile, this supramolecular assembly shows a specific response of fluorescence quenching to Fe^3+^ through the coordination between the sulfonate arms of DSP5 and Fe^3+^ ions, endowing the material with smart cation responsiveness.

## 2. Results

### 2.1. Host–Guest Complexation

DSP5 was successfully designed and synthesized for the first time via a Williamson ether-type synthetic approach ([Supplementary-material supplementary-material-1]). To investigate the host-guest property of DSP5 toward TPE-Q_4_, the following experiments have been conducted. A TPE-based monotopic guest (TPE-Q) with single quaternary ammonium binding site was chosen as a model guest for the study of the host-guest binding between DSP5 and the guest linker moiety on TPE in deionized water at 298 K* via *fluorescence titration ([Supplementary-material supplementary-material-1]). The stoichiometric ratio of DSP5 and the monotopic model guest TPE-Q was determined to be 1:1 and their association constant (*K*_*a*_) was calculated to be (2.68 ± 0.24) × 10^5^ M^−1^. The 2D NOESY spectrum (Figures [Supplementary-material supplementary-material-1] and [Supplementary-material supplementary-material-1]) of the 4:1 mixture of DSP5 (4.0 mM) and TPE-Q_4_ (1.0 mM) in D_2_O showed obvious correlations between the phenyl protons H_a_ of DSP5 and the alkyl chains protons H_1_-H_4_ and methyl protons H_9_ of TPE-Q_4_, indicating the existence of strong host–guest interactions between DSP5 and TPE-Q_4_ and the inclusion of alkyl chains of TPE-Q_4_ into the cavities of DSP5 macrocycles. Furthermore, DOSY experiments were employed to investigate the formation of TPE-Q_4_*⊂* DSP5 in water (see [Supplementary-material supplementary-material-1]). The measured weight-average diffusion coefficient (*D*) of TPE-Q_4_ decreased from 9.66 × 10^−10^ to 8.85 × 10^−10^ m^2^/s with the addition of 4 equiv. of DSP5, suggesting the formation of supramolecular assemblies. Experimental evidence given above has demonstrated the successful construction of the 1:4 supramolecular inclusion complex of TPE-Q_4_*⊂* DSP5.

### 2.2. Fluorescent Properties and Morphology

**Figure 1:**
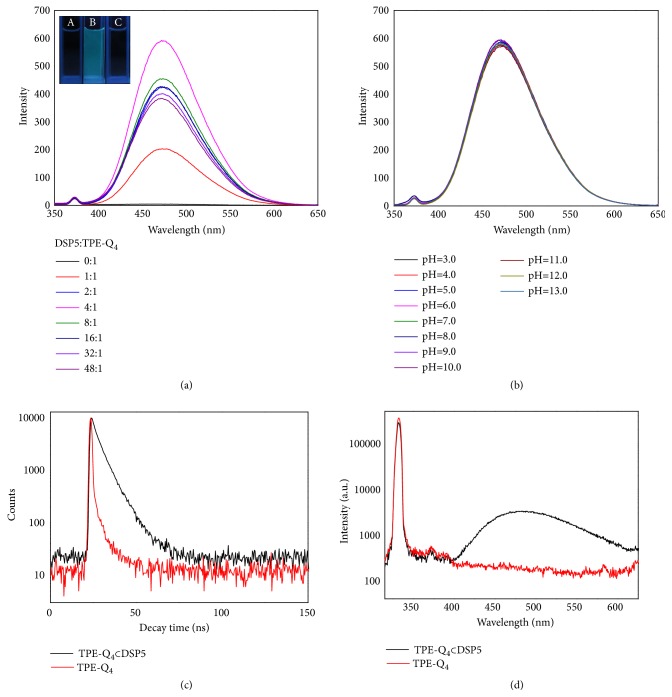
Fluorescence characterizations of TPE-Q_4_ and TPE-Q_4_*⊂* DSP5. (a) Fluorescence spectra of TPE-Q_4_ (1 *μ*M) in water with different concentrations of DSP5 (inset images: (A) DSP5; (B) TPE-Q_4_*⊂* DSP5; (C) TPE-Q_4_ in water); (b) fluorescence spectra of TPE-Q_4_*⊂* DSP5 in PBS of different pH values. Experimental conditions: *λ*_ex_ = 330 nm; *λ*_em_ = 475 nm; slit widths: Ex. 5 nm, Em. 5 nm; 298 K; [TPE-Q_4_] = 1 *μ*M, [DSP5] = 4 *μ*M. (c) Fluorescence decay profiles and (d) absolute fluorescence quantum yields of TPE-Q_4_ and TPE-Q_4_*⊂* DSP5. Experimental conditions: *λ*_ex_ = 365 nm, 298 K, [TPE-Q_4_] = 1 *μ*M, [DSP5] = 4 *μ*M in water.

Fluorescent changes of TPE-Q_4_ (1 *μ*M) in water in the presence of different concentrations of DSP5 were monitored by fluorescence spectroscopy (Figure 1). The emission of TPE-Q_4_ solution is negligible; however upon addition of DSP5, the whole system exhibited green emission color and the fluorescence intensity dramatically increased, reaching the maxima when 4 eq. of DSP5 was added ([DSP5]/[TPE-Q_4_] = 4:1) (Figure 1(a)). The rational explanation is that the intramolecular rotation of phenyl rings of TPE-Q_4_ was restricted by DSP5 upon host-guest inclusion complexation and supramolecular assembly (SAIE). The maximum fluorescence enhancement at the host-guest molar ratio of 4:1 indicated a complete formation of TPE-Q_4_*⊂* DSP5; thus this ratio was chosen for the following studies. Additional supporting data including UV-Vis absorption spectra have also been obtained ([Supplementary-material supplementary-material-1]). It is worth mentioning that negligible changes (Figure 1(b)) could be observed in the fluorescence intensity of TPE-Q_4_*⊂* DSP5 over a wide range of pH (3-13), proving its great pH tolerance and system robustness.

To further investigate the emissive characteristics of TPE-Q_4_*⊂* DSP5, time-resolved fluorescence data of TPE-Q_4_ and TPE-Q_4_*⊂* DSP5 were collected (Figures 1(c) and 1(d), [Supplementary-material supplementary-material-1]). The absolute fluorescence quantum yield of TPE-Q_4_*⊂* DSP5 was 54.40%, which is much higher than that of bare TPE-Q_4_ (0.68%). The photoluminescence decay profiles of TPE-Q_4_ and TPE-Q_4_*⊂* DSP5 suggested that the fluorescence lifetime of TPE-Q_4_ itself is 1.14 ns, while the excited state of TPE-Q_4_*⊂* DSP5 relaxed mainly through a slow pathway with a lifetime of 5.64 ns. Interestingly, TPE-Q_4_*⊂* DSP5 showed one order of magnitude smaller nonradiative decay rate constant (8.08 × 10^7^ s^−1^) and ca. 16 times bigger radiative decay rate constant (9.64 × 10^7^ s^−1^) as compared to bare TPE-Q_4_ (8.68 × 10^8^ s^−1^ and 5.90 × 10^6^ s^−1^, respectively) ([Supplementary-material supplementary-material-1]). These experimental results further support the mechanism of SAIE in the system that the intramolecular motions of the TPE-Q_4_ units are restricted by DSP5 macrocycles, which block the nonradiative relaxation pathway and populated excitons that underwent radiative decay. The above results evidenced that TPE-Q_4_*⊂* DSP5 can emit strong fluorescence even in a dilute solution and DSP5 synthetic macrocycle plays a crucial role in the fluorescence enhancement of this supramolecular assembled system.

**Figure 2:**
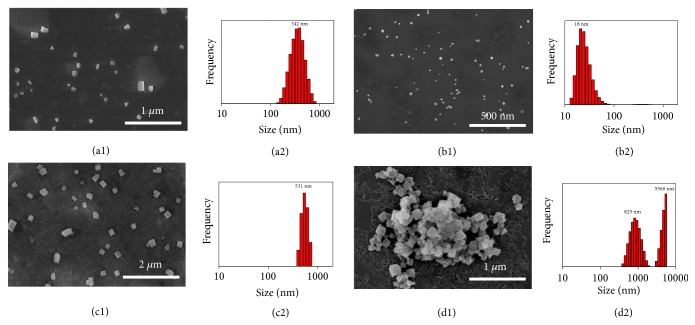
Morphology and size characterizations. SEM images and DLS measurements of (a1) and (a2) TPE-Q_4_, (b1) and (b2) DSP5, (c1) and (c2) TPE-Q_4_*⊂* DSP5, and (d1) and (d2) TPE-Q_4_*⊂* DSP5 with the addition of Fe^3+^ ([TPE-Q_4_] = 1 *μ*M, [DSP5] = 4 *μ*M).

Based on the aforementioned knowledge, we deduced that the amphiphilic nature of TPE-Q_4_ would lead to its self-assembly in aqueous solution, while the ensembles might undergo observable alteration by the host-guest complexation upon addition of DSP5. To obtain direct evidence, the size and morphology of the supramolecular assemblies were studied by scanning electron microscope (SEM) and dynamic light scattering (DLS). Interestingly, cube-shaped assemblies of TPE-Q_4_ with a diameter of ~300 nm and spherical micelles of DSP5 with a diameter of ~20 nm were observed, respectively (Figures 2(a1), 2(a2), 2(b1), and 2(b2)). However, as we envisioned, TPE-Q_4_*⊂* DSP5 exhibited bulky cube-like morphology, yet it possessed a larger diameter of ~500 nm as compared with the nanostructures formed by TPE-Q_4_ or DSP5 alone (Figures 2(c1) and 2(c2)). The formation of supramolecular host-guest complex between DSP5 and TPE-Q_4_ affected the overall amphiphilic nature including the size of building blocks (TPE-Q_4_*⊂* DSP5 versus TPE-Q_4_ or DSP5) and introduced the inter-building block electrostatic repulsions, simultaneously contributing to the increased size of the assembled architectures.

### 2.3. Selective Detection of Fe^*3+*^ Ion

**Figure 3:**
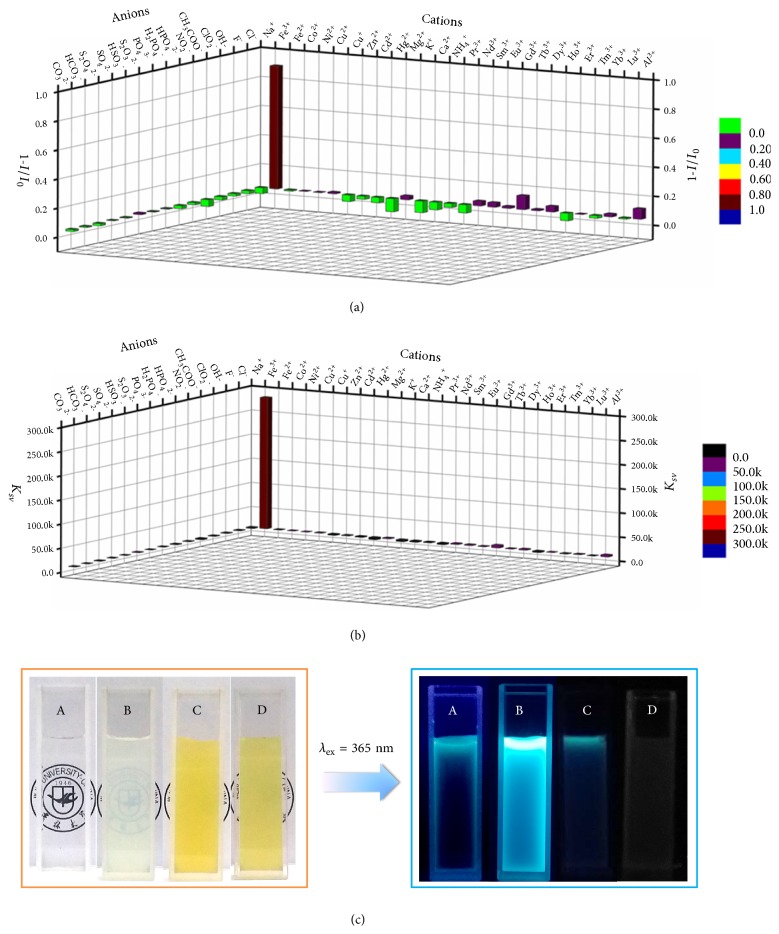
Highly selective detection of Fe^3+^ by TPE-Q_4_*⊂* DSP5. (a) Fluorescence quenching degrees of TPE-Q_4_*⊂* DSP5 in the presence of different cations and anions in water. (b) The Stern–Volmer formula constants of TPE-Q_4_*⊂* DSP5 upon interacting with different cations and anions in water. (c) The pictures of (A) TPE-Q_4_, (B) TPE-Q_4_*⊂* DSP5, (C) TPE-Q_4_*⊂* DSP5 + Fe^3+^, and (D) DSP5 + Fe^3+^ in water. Experimental conditions: *λ*_ex_ = 330 nm; *λ*_em_ = 475 nm; slit widths: Ex. 5 nm, Em. 5 nm; 298 K; [TPE-Q_4_] = 1 *μ*M, [DSP5] = 4 *μ*M, [Fe^3+^] = 20 *μ*M, [Ions] = 20 *μ*M.

Inspired by the above advances, fluorescence experiments were conducted to investigate the responsiveness of TPE-Q_4_*⊂* DSP5 toward different analytes such as ions. Fluorescence quenching degrees and the Stern–Volmer formula constants of TPE-Q_4_*⊂* DSP5 upon addition of different cations and anions at the same concentration were provided (Figures 3(a) and 3(b)), with Cl^−^ and Na^+^ as the counterions, respectively. Surprisingly, only Fe^3+^ ions caused a remarkable fluorescence quenching (1 -* I*/*I*_0_ = 0.84) with a considerably high Stern–Volmer formula constant (*K*_*sv*_ = 2.7 × 10^5^ mol^−1^). To further confirm this ion sensing specificity, experiments on competitive cations were performed and the results showed that fluorescence quenching degrees are as high (~1 -* I*/*I*_0_ > 0.75) with or without competition cations ([Supplementary-material supplementary-material-1]). The limit of detection (LOD) of TPE-Q_4_*⊂* DSP5 to Fe^3+^ was calculated to be 8.6 × 10^−7^ M on the basis of 3*δ*/*S*, indicating that the material possesses a highly sensitive responsiveness to Fe^3+^ ([Supplementary-material supplementary-material-1]). Considering that the material exhibits enhanced fluorescence with good pH stability, the detection of Fe^3+^ by TPE-Q_4_*⊂* DSP5 was further testified under varied pH circumstances, and efficient fluorescence quenching was observed within the wide pH range in the presence of Fe^3+^ ([Supplementary-material supplementary-material-1]), which denoted that the material can be applied for the selective detection of Fe^3+^ in aqueous conditions without the interference of pH levels.

### 2.4. Investigation of the Quenching Mechanism

To further explore the quenching mechanism, a series of measurements were carried out. SEM image of TPE-Q_4_*⊂* DSP5 in the presence of Fe^3+^ showed irregular morphology, which was different from the assembly structure of TPE-Q_4_*⊂* DSP5 itself (Figures 2(d1) and 2(d2)); thus we consider that a complexation between DSP5 and Fe^3+^ occurred. In order to exclude the influence of the electron-rich cavity of DSP5, monomer molecule (DSNa) with sulfonate groups was synthesized. SEM study demonstrated that Fe^3+^ could coordinate with the sulfonate groups of DSNa ([Supplementary-material supplementary-material-1]). Moreover, ^1^H NMR experiments were carried out to confirm the coordination of DSP5 and Fe^3+^ ([Supplementary-material supplementary-material-1]). Because TPE-Q_4_*⊂* DSP5 precipitated from D_2_O in the NMR tube, no peaks could be observed in the ^1^H NMR spectrum. However, the proton peaks of TPE-Q_4_ reappeared after the addition of Fe^3+^ but the proton peaks of DSP5 were still absent in the spectrum, suggesting that DSP5 coordinated with Fe^3+^, forming water-insoluble coordination polymers. Fluorescent images were also obtained to further confirm the existence of TPE-Q_4_ in the aqueous media, where the fluorescence recovered when DSP5 was added after the precipitation ([Supplementary-material supplementary-material-1]).

The ion-responsive property of TPE-Q_4_*⊂* DSP5 and the interactions of DSP5 and Fe^3+^ were described in a more intuitive way in Figure 3(c). The TPE-Q_4_*⊂* DSP5 water solution (B) was clear and emitted intensely under 365 nm irradiation, yet precipitation and quenching occurred after adding Fe^3+^ (C) at a higher concentration. After centrifugation, the precipitates were separated from the solutions ([Supplementary-material supplementary-material-1]). However, A and B solutions still showed Tyndall effect but C and D solutions did not, indicating larger supramolecular assemblies in C and D than in A and B solutions. Importantly, the precipitation in D further confirmed that DSP5 interacted with Fe^3+^, leaving TPE-Q_4_ free in water, where the fluorescence was greatly quenched.

**Figure 4:**
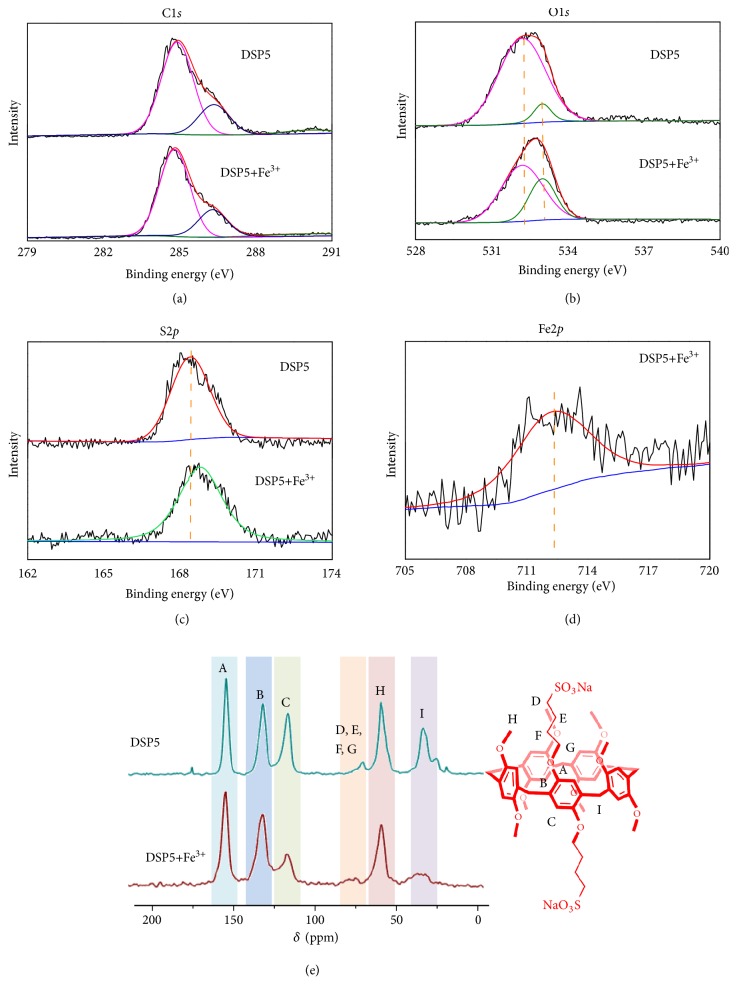
Coordination effect between DSP5 and Fe^3+^. (a) C1s, (b) O1s, and (c) S2p regions of XPS spectra of DSP5 before (up) and after (down) the addition of Fe^3+^, and (d) Fe2p regions of XPS spectrum of DSP5+Fe^3+^ powder. (e) Solid-state CP-MAS ^13^C NMR characterization of DSP5 and DSP5 + Fe^3+^ powder.

To prove the coordination effect between sulfonate groups of DSP5 and Fe^3+^, we used X-ray photoelectron spectroscopy (XPS) to record the valence state changes of DSP5 before and after the addition of Fe^3+^ (Figures 4(a)–4(d)). The XPS spectra demonstrated that the binding energies of the C1s peaks at 284.7 eV, 290.0 eV, and 286.2 eV underwent no obvious changes. But the binding energy of the S2p peak at 168.4 eV, attributing to the sulfonate group, changed to 168.8 eV upon the addition of Fe^3+^ [31]. Moreover, the binding energy of Fe2p (712.2 eV) is different from the ferric chloride (711.5 eV) according to previous literature [32]. Besides, solid-state CP-MAS ^13^C NMR of the DSP5 and DSP5+Fe^3+^ powder also provided convincing evidences for the coordination of DSP5 and Fe^3+^ (Figure 4(e)).

## 3. Discussion

In conclusion, we have successfully synthesized a novel water-soluble disulfonate-functionalized pillar[5]arene (DSP5), based on which a pH-stable fluorescent material (TPE-Q_4_*⊂* DSP5) was facilely designed and constructed, exhibiting SAIE properties. The as-prepared supramolecular material showed excellent photoluminescent characteristics of emitting switchable bright blue fluorescence even in dilute aqueous solution. In addition, the supramolecular assembly possessed superior responsiveness to Fe^3+^ in water with high selectivity and sensitivity, since the enhanced fluorescence arising from TPE-Q_4_*⊂* DSP5 could be efficiently quenched by the coordination effect of DSP5 and Fe^3+^. We envision that this new type of supramolecular fluorescent materials will find great applications in various fields, including fluorescent probing, cell imaging, and visualized monitoring of metal ions.

## 4. Materials and Methods

### 4.1. Materials and Characterization

All reagents were commercially available and used as supplied without further purification. ^1^H NMR spectra were collected on a temperature-controlled 300 MHz spectrometer. ^13^C NMR spectra were recorded on a DMX-500 spectrometer. Solid-state cross-polarization magic angle spinning (CP/MAS) ^13^C NMR spectra of polymers were measured on a Bruker Digital Avance III HD 400 WB (400 MHz) NMR spectrometer at ambient temperature with a magic angle spinning rate of 7.0 kHz internal reference. 2D diffusion-ordered NMR spectroscopy (DOSY) and 2D nuclear Overhauser effect NMR spectroscopy (NOESY) were recorded on a Bruker Avance DMX 600 spectrophotometer. High-resolution electrospray ionization mass spectra (HRESI-MS) were obtained on a Bruker 7-Tesla FT-ICR mass spectrometer equipped with an electrospray source (Billerica, MA, USA). The UV-vis spectra were collected via UV-vis spectroscopy on a Shimadzu UV-1800 spectrophotometer. Deionized water, purifying by Experimental Water System (Lab-UV-20), was used in relevant experiments. Scanning electron microscope (SEM) investigations were carried out on a HITACHI-SU8020 instrument. Dynamic light scattering (DLS) results were obtained on a Zetasizer Nano ZS instrument. The fluorescence experiments were conducted on a RF-5301 spectrofluorometer (Shimadzu Corporation, Japan). The surface compositions of polymers were determined by X-ray photoelectron spectroscopy (XPS) measured on a PREVAC XPS/UPS System using monochromatic Al *K*_*α*_ (1486.7 eV) radiation. The time-resolved fluorescence decay curves and absolute fluorescence quantum yields were obtained on a FLS920 instrument (Edinburgh Instrument) with the excitation of 330 nm.

### 4.2. Syntheses of Compound

The detailed synthesis procedures and characterizations of the studied compound (DSP5, TPE-Q_4_, TPE-Q, and DSNa) can be found in the Supplementary Materials.

### 4.3. Detection of Fe^*3+*^

Experiments were carried out to examine the potential for detecting cations and anions. Firstly, we prepared the DSP5 and TPE-Q_4_*⊂* DSP5 aqueous solution ([TPE-Q_4_] = 4 *μ*M, [DSP5] = 1 *μ*M). 40 *μ*L M(Cl)_x_ aqueous solution (M^x+^ = Na^+^, Fe^3+^, Fe^2+^, Co^2+^, Ni^2+^, Cu^2+^, Cu^+^, Zn^2+^, Cd^2+^, Hg^2+^, Mg^2+^, K^+^, Ca^2+^, NH_4_^+^, Pr^3+^, Nd^3+^, Sm^3+^, Eu^3+^, Gd^3+^, Tb^3+^, Dy^3+^, Ho^3+^, Er^3+^, Tm^3+^, Yb^3+^, Lu^3+^, and Al^3+^, [M^x+^] = 2 mM) and 40 *μ*L (Na)_y_N aqueous solution (N^y-^ = Cl^−^, F^−^, OH^−^, ClO^−^, CH_3_COO^−^, NO_2_^−^, HPO_4_^2−^, H_2_PO_4_^−^, PO_4_^3−^, S_2_O_3_^2−^, HSO_3_^−^, SO_4_^2−^, S_2_O_4_^2−^, HCO_3_^−^, CO_3_^2−^, [N^y-^] = 2 mM) were added into the above TPE-Q_4_*⊂* DSP5 solution (3.96 mL), respectively. Then, the fluorescence of each solution was recorded immediately. Similarly, interference metal ion with the same concentration as Fe^3+^ was added into the TPE-Q_4_*⊂* DSP5 solution containing Fe^3+^ to further investigate the fluorescence recognition of Fe^3+^. The fluorescence quenching induced by Fe^3+^ was investigated to confirm whether the developed TPE-Q_4_*⊂* DSP5 could be used to quantitative detection of Fe^3+^. Thus, different volumes of Fe^3+^ solutions (1 mM, 0~28 *μ*L) were added into TPE-Q_4_*⊂* DSP5 solution (1 *μ*M) and the fluorescence intensities were recorded, respectively.

### 4.4. Preparation of DSP5 + Fe^*3+*^ Powder

FeCl_3_ aqueous solution (1 mL, 0.1 mM) was added to DSP5 aqueous solution (0.1 mM, 8 mL) and the precipitates were generated immediately. Let the mixture stood for about 24 h and the precipitates were isolated by centrifuge at 10000 rpm. Then the product was washed with deionized water for three times to remove the FeCl_3_ residue and dried under high vacuum oven to get a brown powder.

## Supplementary Materials

Supplementary Materials Scheme S1. Synthetic route of the targeted compound DSP5. Scheme S2. Synthetic route of the targeted compound TPE-Q_4_. Scheme S3. Synthetic route of the targeted compound TPE-Q. Scheme S4. Synthetic route of the targeted compound DSNa. Figure S1. The study of (a) and (b) association constant (*K*_a_), and (c) stoichiometric ratio between DSP5 and TPE-Q. Figure S2. NOESY spectrum (600 MHz, D_2_O, 298 K) of a mixture of TPE-Q_4_ (1.0 mM) and DSP5 (4.00 mM). Figure S3. Partial NOESY spectrum (600 MHz, D_2_O, 298 K) of a mixture of TPE-Q_4_ (1.0 mM) and DSP5 (4.00 mM). Figure S4. DOSY spectrum (600 MHz, D_2_O, 298 K) of (a) TPE-Q_4_ (1.00 mM) and (b) a mixture of TPE-Q_4_ (1.0 mM) and DSP5 (4.00 mM). Figure S5. UV-vis spectra of TPE-Q_4_ (1 *µ*M) in water with different concentrations of DSP5. Figure S6. Fluorescence quenching degrees in competition experiments of TPE-Q_4_*⊂* DSP5 in water. Front: TPE-Q_4_*⊂* DSP5 with different competing cations; back: TPE-Q_4_*⊂* DSP5 with Fe^3+^ and the same equiv of cations. (Experimental conditions: *λ*_ex_ = 330 nm; *λ*_em_ = 475 nm; slit widths: Ex. 5 nm, Em. 5 nm; 298 K; [TPE-Q_4_] = 1 *µ*M, [DSP5] = 4 *µ*M, [Fe^3+^] = 20 *µ*M, [Ions] = 20 *µ*M). Figure S7. Fluorescence quenching degrees and the Stern–Volmer formula constants of TPE-Q_4_*⊂* DSP5 in the presence of different cations and anions in water. 2D plots for all the M^x+^ with Cl^−^ (a, c) and Na^+^ with N^y−^ (b, d). Figure S8. (a) Fluorescence spectra of supramolecular assemblies TPE-Q_4_*⊂* DSP5 aqueous solution in the presence of increasing amounts of Fe^3+^; (b) the photograph of the linear range (experimental conditions: *λ*_ex_ = 330 nm; *λ*_em_ = 475 nm; slit widths: Ex. 5 nm, Em. 5 nm; 298 K; [TPE-Q_4_] = 1 *µ*M, [DSP5] = 4 *µ*M). Figure S9. Fluorescence spectra of TPE-Q4 *⊂* DSP5 in the presence of Fe^3+^ under different pH values. Figure S10. SEM images of (a) DSP5+Fe^3+^ and (b) DSNa+Fe^3+^; TEM images of (c) DSP5+Fe^3+^ and (d) TPE-Q_4_*⊂* DSP5+Fe^3+^ ([DSP5] = 10 *µ*M, [DSNa] = 50 *µ*M, [DSP5]/[Fe^3+^] = 1:20, [DSNa]/[Fe^3+^] = 1:20). Figure S11. ^1^H NMR spectra of TPE-Q_4_, TPE-Q_4_*⊂* DSP5 and TPE-Q_4_*⊂* DSP5+Fe^3+^ ([TPE-Q_4_] = 1.00 mM, [DSP5] = 4.00 mM, [Fe^3+^] = 20.00 mM, 300 MHz, D_2_O, 298 K). Figure S12. The pictures of (A) TPE-Q_4_, (B) TPE-Q_4_*⊂* DSP5, (C) TPE-Q_4_*⊂* DSP5 + Fe^3+^ and (D) supernatant upon addition of DSP5 again after removing the DSP5 + Fe^3+^ precipitate. Figure S13. The pictures of (A) TPE-Q_4_, (B) TPE-Q_4_*⊂* DSP5, (C) TPE-Q_4_*⊂* DSP5 + Fe^3+^, and (D) DSP5 + Fe^3+^ in water (after centrifugation). Figure S14. ^1^H NMR spectrum (300 MHz, CDCl_3_, 298 K) of compound 1. Figure S15. ^1^H NMR spectrum (300 MHz, CDCl_3_, 298 K) of compound 2. Figure S16. ^1^H NMR spectrum (300 MHz, CDCl_3_, 298 K) of compound 3. Figure S17. ^13^C NMR spectrum (126 MHz, D_2_O, 298 K) of DSP5. Figure S18. ^1^H NMR spectrum (300 MHz, D_2_O, 298 K) of DSP5. Figure S19. HRESI-MS m/z [M-Na]^−^ calcd. for C_51_H_60_O_16_S_2_Na^−^ 1015.3215, found 1015.3226. Figure S20. ^1^H NMR spectrum (300 MHz, DMSO-*d*_*6*_, 298 K) of compound 4. Figure S21. ^1^H NMR spectrum (300 MHz, D_2_O, 298 K) of TPE-Q_4_. Figure S22. ^1^H NMR spectrum (300 MHz, CDCl_3_, 298 K) of compound 5. Figure S23. ^1^H NMR spectrum (300 MHz, CDCl_3_, 298 K) of compound 6. Figure S24. ^1^H NMR spectrum (300 MHz, CDCl_3_, 298 K) of compound 7. Figure S25. ^1^H NMR spectrum (300 MHz, DMSO-*d*_*6*_, 298 K) of TPE-Q. Figure S26. ^1^H NMR spectrum (300 MHz, D_2_O, 298 K) of DSNa. Table S1. Emission lifetimes (*τ*), radiative decay rate constant (*k*_rad_), and nonradiative decay rate constant (*k*_nrad_) of bare TPE-Q_4_ and TPE-Q_4_*⊂* DSP5 in water.
