# Supplementary material for: A Binary Supramolecular Assembly with Intense Fluorescence Emission, High pH Stability, and Cation Selectivity: Supramolecular Assembly-Induced Emission Materials
Source: Research (Wash D C). 2019 Jul 24;2019:1454562. doi: 10.34133/2019/1454562 (PMC6750118; doi:10.34133/2019/1454562)
Supplement: Supplementary Materials — Scheme S1. Synthetic route of the targeted compound DSP5. Scheme S2. Synthetic route of the targeted compound TPE-Q4. Scheme S3. Synthetic route of the targeted compound TPE-Q. Scheme S4. Synthetic route of the targeted compound DSNa. Figure S1. The study of (a) and (b) association constant (Ka), and (c) stoichiometric ratio between DSP5 and TPE-Q. Figure S2. NOESY spectrum (600 MHz, D2O, 298 K) of a mixture of TPE-Q4 (1.0 mM) and DSP5 (4.00 mM). Figure S3. Partial NOESY spectrum (600 MHz, D2O, 298 K) of a mixture of TPE-Q4 (1.0 mM) and DSP5 (4.00 mM). Figure S4. DOSY spectrum (600 MHz, D2O, 298 K) of (a) TPE-Q4 (1.00 mM) and (b) a mixture of TPE-Q4 (1.0 mM) and DSP5 (4.00 mM). Figure S5. UV-vis spectra of TPE-Q4 (1 µM) in water with different concentrations of DSP5. Figure S6. Fluorescence quenching degrees in competition experiments of TPE-Q4⊂ DSP5 in water. Front: TPE-Q4⊂ DSP5 with different competing cations; back: TPE-Q4⊂ DSP5 with Fe3+ and the same equiv of cations. (Experimental conditions: λex = 330 nm; λem = 475 nm; slit widths: Ex. 5 nm, Em. 5 nm; 298 K; [TPE-Q4] = 1 µM, [DSP5] = 4 µM, [Fe3+] = 20 µM, [Ions] = 20 µM). Figure S7. Fluorescence quenching degrees and the Stern–Volmer formula constants of TPE-Q4⊂ DSP5 in the presence of different cations and anions in water. 2D plots for all the Mx+ with Cl− (a, c) and Na+ with Ny− (b, d). Figure S8. (a) Fluorescence spectra of supramolecular assemblies TPE-Q4⊂ DSP5 aqueous solution in the presence of increasing amounts of Fe3+; (b) the photograph of the linear range (experimental conditions: λex = 330 nm; λem = 475 nm; slit widths: Ex. 5 nm, Em. 5 nm; 298 K; [TPE-Q4] = 1 µM, [DSP5] = 4 µM). Figure S9. Fluorescence spectra of TPE-Q4 ⊂ DSP5 in the presence of Fe3+ under different pH values. Figure S10. SEM images of (a) DSP5+Fe3+ and (b) DSNa+Fe3+; TEM images of (c) DSP5+Fe3+ and (d) TPE-Q4⊂ DSP5+Fe3+ ([DSP5] = 10 µM, [DSNa] = 50 µM, [DSP5]/[Fe3+] = 1:20, [DSNa]/[Fe3+] = 1:20). Figure S11. 1H NMR spectra of TPE- [file 1454562.f1.pdf]

Supplementary Materials

A Binary Supramolecular Assembly with Intense Fluorescence Emission, High pH Stability, and Cation Selectivity

Xu Wang[1], Xin-Yue Lou[1], Xiao-Yu Jin[1], Feng Liang[2], and Ying-Wei Yang[1,2]*

[1]State Key Laboratory of Inorganic Synthesis and Preparative Chemistry, International Joint Research Laboratory of Nano-Micro Architecture Chemistry, College of Chemistry, Jilin University,2699 Qianjin Street, Changchun 130012, P. R. China.

[2]The State Key Laboratory of Refractories and Metallurgy, School of Chemistry & Chemical Engineering, Wuhan University of Science and Technology, Wuhan 430081, P. R. China

*Correspondence should be addressed to Ying-Wei Yang; ywyang@jlu.edu.cn.

1. Experimental section

1.1 Synthesis of DSP5

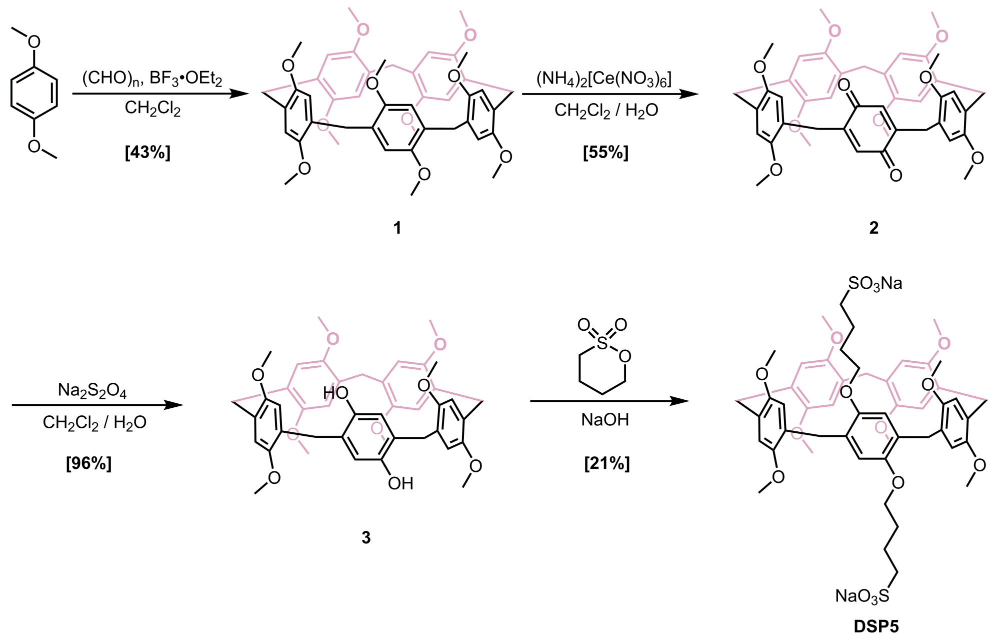

Scheme S1. Synthetic route of the targeted compound **DSP5**.

Synthesis of compound 1

1, 4-Dimethoxybenzene (7g, 50 mmol) and $(CH_2O)_n$ (4.5g, 150 mmol) were dissolved in dry CH_2Cl_2 (500 mL) in a 1000 mL flask and stirred at 0 °C for 30 min. Then $BF_3{\cdot}OEt_2$ (7 mL) was added into the flask carefully. The mixture was reacted for 30 min and the reaction was quenched with $NaHCO_3$ aqueous solution and extracted with CH_2Cl_2 for three times. The organic layers were combined and dried over magnesium sulfate, then concentrated under vacuum after filtration. The crude product was further purified by silica gel column chromatography (petroleum ether/dichloromethane, 1:2 *v*/*v* as the eluent) to get a white powder (3.2 g, 43%). ^{1}H NMR (300 MHz, $CDCl_3$, 298 K): δ (ppm): 6.759 (10 H, s), 3.773 (10 H, s), 3.643 (30 H, s).

Synthesis of compound 2

Compound **1** (3g, 4mmol) was dissolved in CH_2Cl_2 (200 mL) in a 500 mL flask and stirred at room temperature. Then a solution of CAN (4.38g, 8mmol) was added into the flask dropwise. After stirring for 1 h, the reaction was stopped and the mixture was extracted with CH_2Cl_2 and washed with NaCl aqueous solution for three times. The organic layer was concentrated under vacuum and subjected to silica gel chromatography (petroleum ether/dichloromethane, 1:1 *v*/*v* as the eluent) to get a red powder (1.6 g, 55%) .^{1}H NMR (300 MHz, $CDCl_3$, 298 K): δ (ppm): 6.845-6.670 (10 H, m), 3.786-3.590 (34 H, m).

Synthesis of compound 3

Compound **2** (500 mg, 1 mmol) was dissolved in CH_2Cl_2 (10 mL) in a 50 mL flask under N_2 atmosphere. Then a solution of $Na_2S_2O_4$ (11.2 g, 7 mmol) was added into the flask dropwise. After stirring for 1 h, the reaction was stopped and the mixture was extracted with CH_2Cl_2 and washed with NaCl aqueous solution for three times. The organic layer was concentrated under vacuum to get a white powder (480 mg, 96%). ^{1}H NMR (300 MHz, $CDCl_3$, 298 K): δ (ppm): 7.175 (2 H, s), 6.589-6.906 (10 H, m), 3.645-3.837 (34 H, m).

Synthesis of DSP5

Compound **3** (480 mg, 0.6 mmol) and NaOH (115 mg, 3 mmol) were added into THF (30 mL) in a 50 mL flask. Then 1, 4-butylenesulfone (0.24 mL) was added into the above solution and the mixture reacted at room temperature for 24 h. Then filtered to collect the crude product and the crude was washed with THF and CH_2Cl_2. The product was dried under high vacuum to get a brown powder (177 mg, 21%). ^{1}H NMR (300 MHz, D_2O, 298 K): δ (ppm): 6.683-6.552 (m, 10 H), 3.973-3.348 (m, 38 H), 2.830 (m, 4 H), 1.770~1.566 (m, 8 H); ^{13}C NMR (126 MHz, D_2O, 298 K): δ (ppm): 150.895, 150.245, 128.635, 114.281, 68.662, 55.611, 50.762, 30.072, 27.905, 21.230; HRMS (ESI) *m*/*z*: $[M-Na]^-$ calcd. for $C_{51}H_{60}O_{16}S_2Na^-$, 1015.3215; found: 1015.3226.

1.2 Synthesis of TPE-Q$_4$

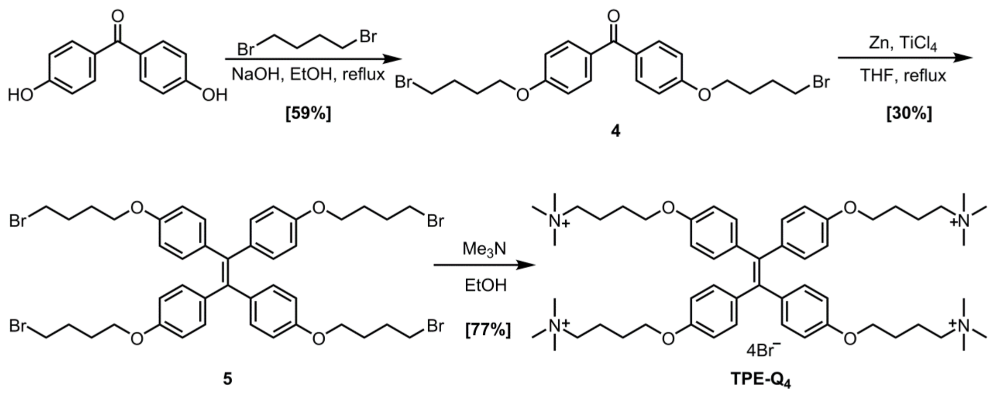

Scheme S2. Synthetic route of the targeted compound **TPE-Q$_4$**.

Synthesis of compound 4

A solution of bis(4-hydroxyphenyl)methanone (5 g, 23.4 mmol) and NaOH (3.7 g, 56 mmol) in CH_3CH_2OH (250 mL) was stirred in a 500 mL round bottom flask. Then 1, 4-dibromobutane (16.7 mL) was added into the above solution and the mixture reacted at reflux temperature for 12 h. The crude product was washed with CH_3CH_2OH and water and dried under high vacuum oven to get a white powder (6.7g, 59%). ^{1}H NMR (300 MHz, DMSO-d_6, 298 K): δ (ppm): 7.644 (d, 4 H), 7.079 (d, 4 H), 4.121 (t, 4 H), 3.628 (t, 4 H), 2.035-1.827 (m, 8 H).

Synthesis of compound 5

Znic (1.7 g) and titanium tetrachloride (1.7 mL) were added into dry tetrahydrofuran (100 mL) at 0 °C and the mixture was stirred for 30 min. Then compound **4** (2.4 g) was added into above solution and reacted at reflux temperature for 3 h. The crude product was separated, extracted and concentrated. The obtained product was subjected to column chromatograph (petroleum ether/dichloromethane, 2:1 *v*/*v*) to get a white powder (550 mg, 30%). ^{1}H NMR (300 MHz, $CDCl_3$, 298 K): δ (ppm): 6.890 (d, 8 H), 6.610 (d, 8 H), 3.913 (t, 8 H), 3.477 (t, 8 H), 2.094-1.854 (m, 16 H).

Synthesis of TPE-Q$_4$

Compound **5** (500 mg, 0.6 mmol) and trimethylamine (33% in ethanol, 7.2 mL, 28 mmol) were added to ethanol (60 mL). The solution was stirred at reflux temperature overnight. Then the solvent was removed by evaporation and deionized water (25 mL) was added. After filtration, a clear solution was got. Then water was removed by evaporation to get a light green powder (430 mg, 77%). ^{1}H NMR (300 MHz, D_2O, 298 K) δ (ppm): 6.972 (d, 8H), 6.561 (d, 8H), 3.664 (t, 8H), 3.298 (m, 8H), 3.067 (s, 36H), 1.719-1.631 (m, 16H).

1.3 Synthesis of TPE-Q

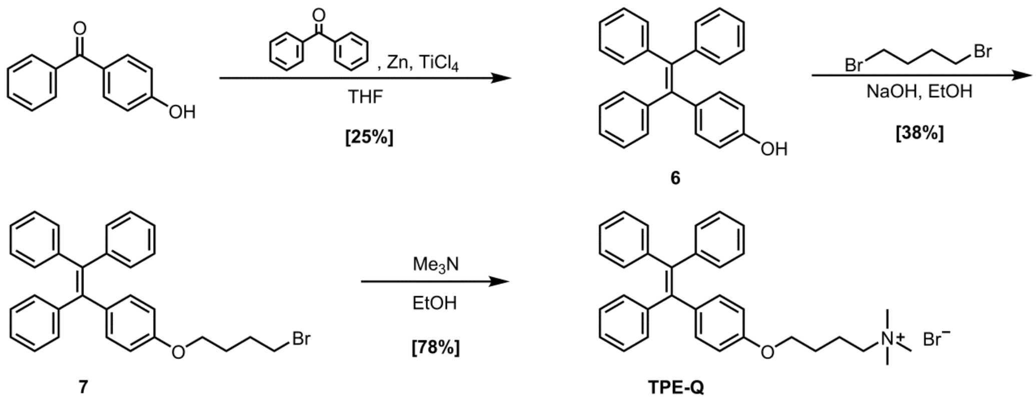

Scheme S3. Synthetic route of the targeted compound **TPE-Q**.

Synthesis of compound 6

Znic (6.8 g) and titaniumtetrachloride (6.8 mL) was added into dry tetrahydrofuran (240 mL) at 0 °C and the mixture was reacted at 0 °C for 30 min. Then 4-hydroxybenzophenone (1.98 g) and benzophenone (1.82 g) tetrahydrofuran solution was added into the above solution and the mixture was stirred at reflux temperature for 18 h. Then the mixture was added with potassium carbonate solution to quench the reaction. The crude product was separated, extracted and concentrated. The obtained product was subjected to column chromatograph (petroleum ether/dichloromethane, 1:2 *v*/*v*) to get a yellow powder (860 mg, 25%). ^{1}H NMR (300 MHz, $CDCl_3$, 298 K) δ (ppm): 7.049-6.971 (m, 15H), 6.853 (d, 2H), 6.505 (d, 2H).

Synthesis of compound 7

Compound **6** (160 mg, 4.6 mmol) and K_2CO_3 (1 g, 7.5 mmol) were added into CH_3CN (80 mL). The mixture was stirred for 60 min at room temperature. Then 1, 4-dibromobutan (1 mL, 7.5 mmol) was added into the mixture. The mixture was stirred at reflux temperature for 24 h. The obtained product was subjected to column chromatograph (petroleum ether/dichloromethane, 2:1 *v*/*v*) to get a yellow powder (60 mg, 38%). ^{1}H NMR (300 MHz, $CDCl_3$, 298 K) δ (ppm): 7.054-6.905 (m, 17H), 6.613 (d, 2H), 3.914 (t, 2H), 3.472 (t, 2H), 2.085-1.851 (m, 4H).

Synthesis of TPE-Q

Compound **7** (100 mg, 0.2 mmol) and trimethylamine (0.5 mL) were added into ethanol (10 mL). The mixture was stirred at 90 °C for 24 h. Then the mixture was washed with CH_2Cl_2. The product was dried under high vacuum to get a yellow powder (80 mg, 78%). ^{1}H NMR (300 MHz, DMSO-d_6, 298 K) δ (ppm): 7.159-6.688 (m, 19H), 3.928 (t, 2H), 3.101-3.044 (m, 11H), 1.807-1.683 (m, 4H).

1.4 Synthesis of DSNa

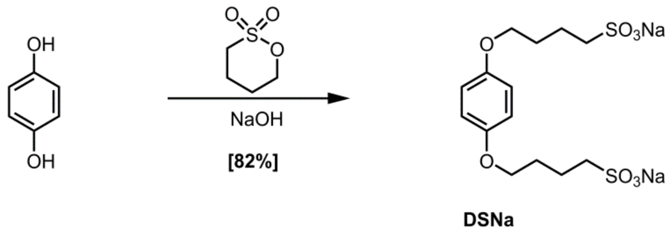

Scheme S4. Synthetic route of the targeted compound **DSNa**.

A solution of butanesultone (12.0 g, 100 mmol) in 1, 4-dioxane (100 mL) was added into a solution of hydroquinone (4.4 g, 40.0 mmol) in aqueous NaOH solution (10 wt%, 60 mL). The mixture was stirred at room temperature for 12 h and filtered to collect the crude solid. The solid was stirred in acetone (100 mL) and dried under high vacuum to get a white solid (12.9 g, 82%).^{1}H NMR (300 MHz, D_2O, 298 K): 6.999 (s, 4H), 4.071 (t, 4H), 2.980 (t, 4H), 1.972-1.881 (m, 8H).

2. Host-guest interaction investigation

To quantitatively assess the inclusion complexation behavior, fluorescence titrations of DSP5 and TPE-Q guest were performed at 298 K in deionized water. Using a nonlinear least-squares curve-fitting method, the association constant was obtained for each host-guest combination from the following equation.

ΔI=0.5*(alpha)*((G_0/2+H_0+(1/K_a))-(sqrt((G_0/2+H_0+(1/K_a))*(G_0/2+H_0+(1/K_a))-4*G_0/2*H_0)))

Where ΔI is the fluorescence intensity change of guest at $[H]_0$ compared with at $[G]_0$, $[G]_0$ is the fixed initial concentration of the guest, and $[H]_0$ is the initial concentration of host.

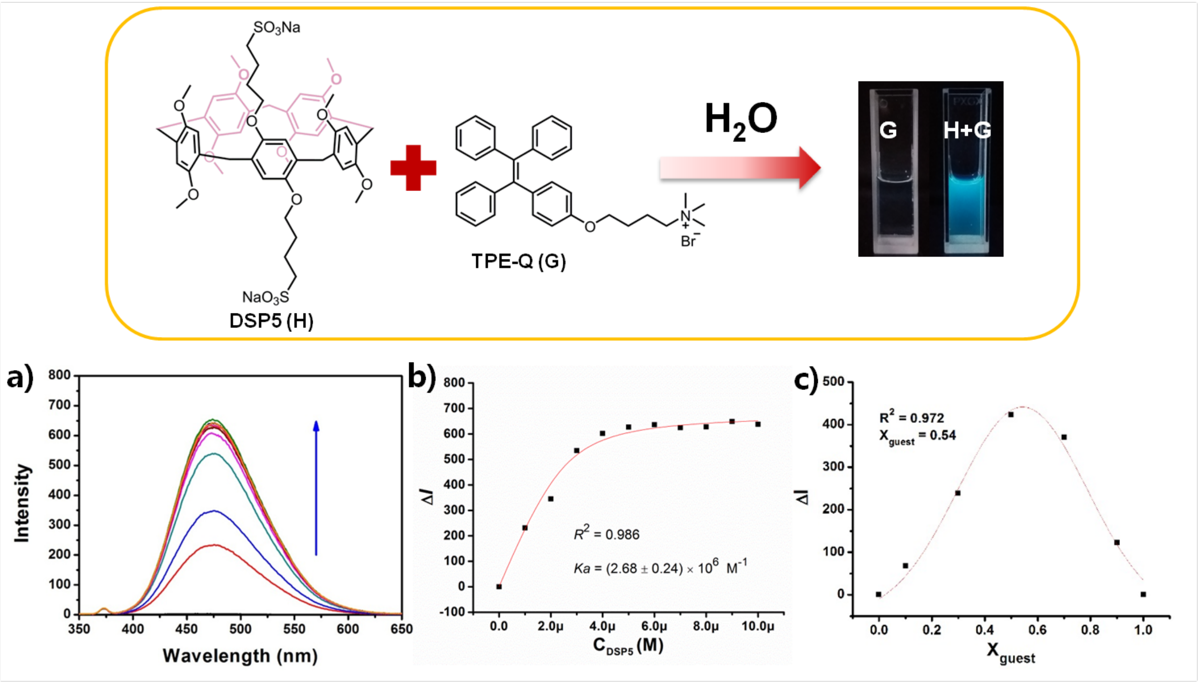

Figure S1. The study of a) and b) association constant (K_a), and c) stoichiometric ratio between DSP5 and TPE-Q.

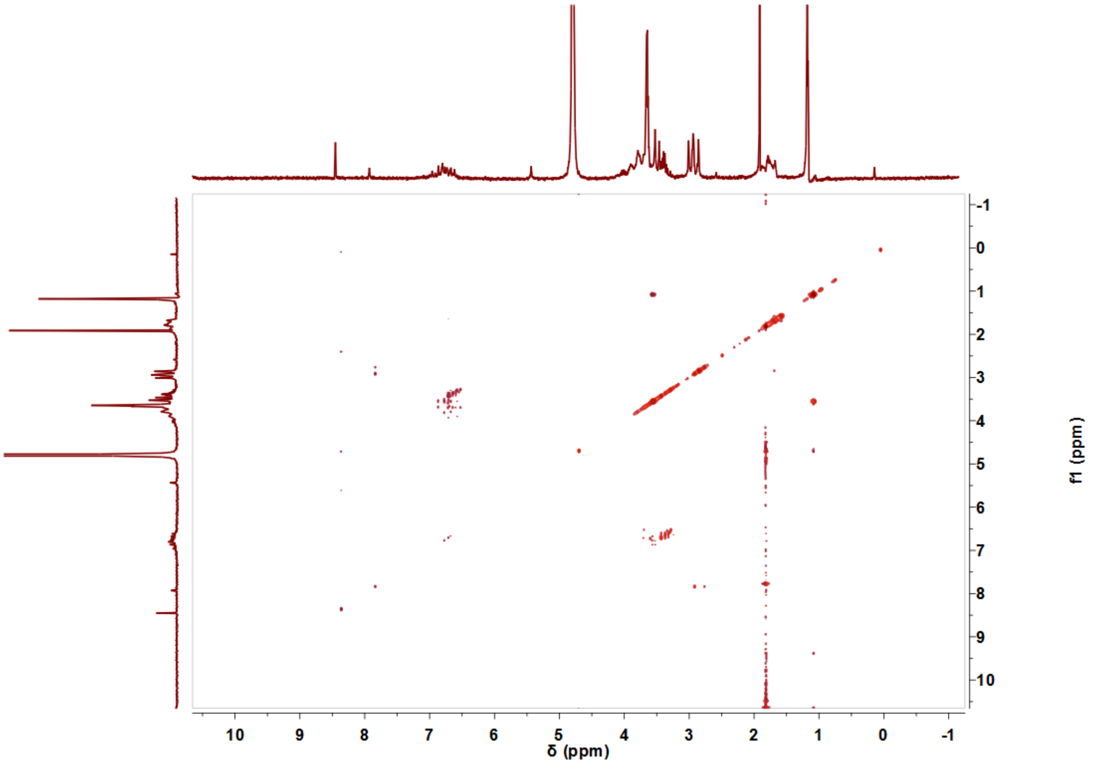

Figure S2. NOESY spectrum (600 MHz, D_2O, 298 K) of a mixture of TPE-Q_4 (1.0 mM) and DSP5 (4.00 mM).

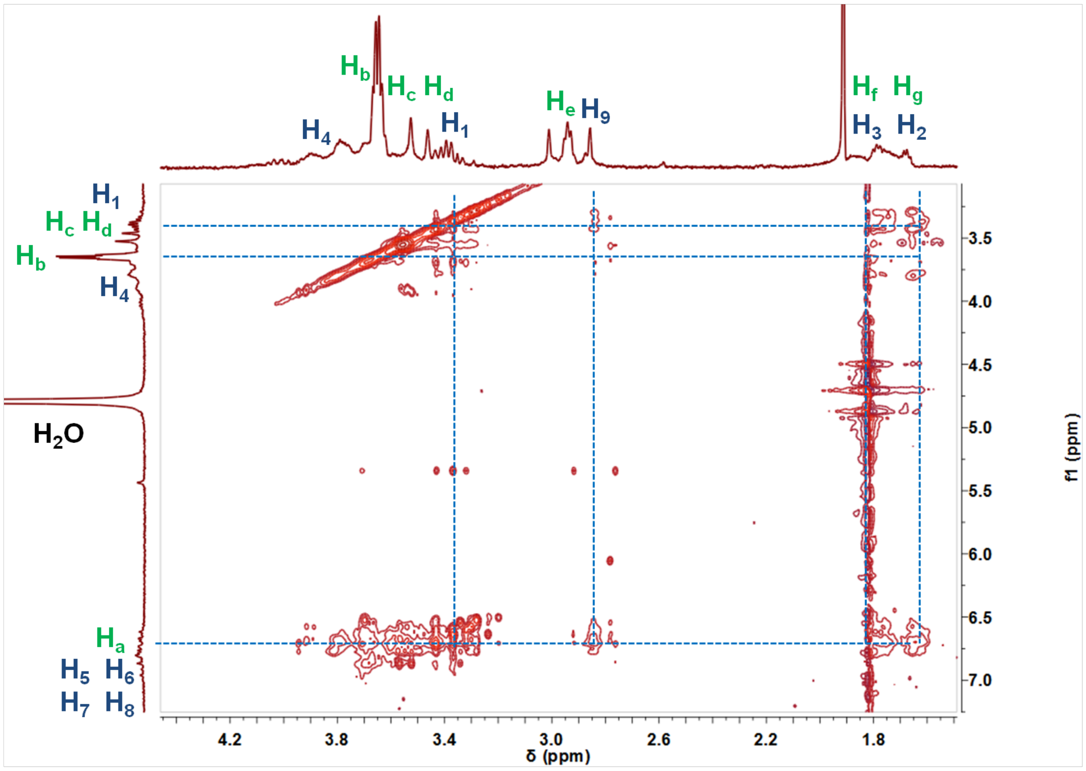

Figure S3. Partial NOESY spectrum (600 MHz, D_2O, 298 K) of a mixture of TPE-Q_4 (1.0 mM) and DSP5 (4.00 mM).

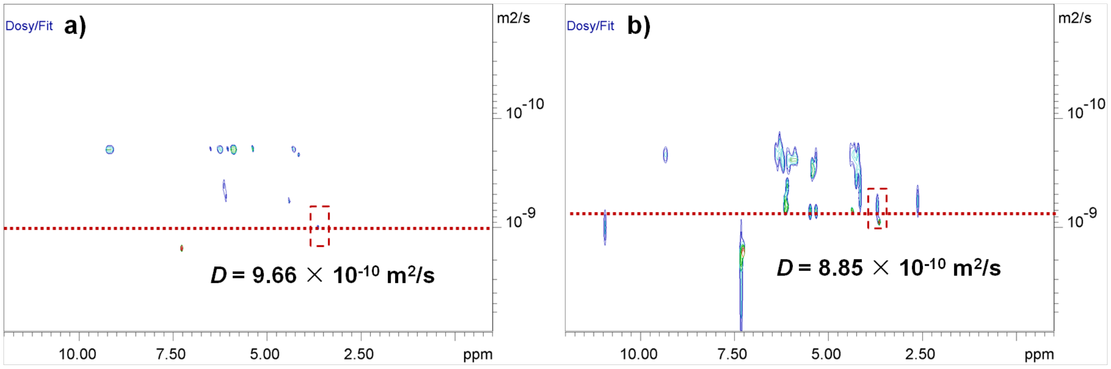

Figure S4. DOSY spectrum (600 MHz, D_2O, 298 K) of a) TPE-Q_4 (1.00 mM) and b) a mixture of TPE-Q_4 (1.0 mM) and DSP5 (4.00 mM).

3. UV-vis characterizations

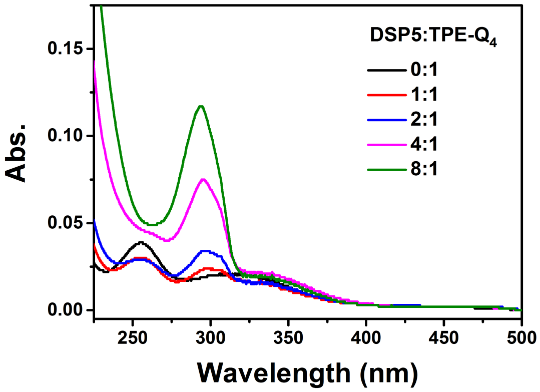

Figure S5. UV-vis spectra of TPE-Q_4 (1 μM) in water with different concentrations of DSP5.

4. Calculation of radiative and non-radiative decay rate constants

Fluorescence typically follows first-order kinetics:

$$[S] = [S]_0 e^{-t/\tau} \qquad (1)$$

[S] is the concentration of exited state molecules at time t, $[S]_0$ is the initial concentration and is the fluorescence lifetime.

Decay rate (k) is the inverse of lifetime, consisting of radiative and non-radiative decay rate constants:

$$k = k_{rad} + k_{nrad} \qquad (2)$$

where k_{rad} is the radiative decay rate constant and k_{nrad} is the nonradiative decay rate constant. The quantum yield (QE) is defined as the fraction of emission process in which emission of light is involved:

$$QE = k_{rad} / (k_{rad} + k_{nrad}) \qquad (3)$$

The values of radiative and non-radiative rate constants of bare TPE-Q_4 and TPE-Q_4⊂DSP5 were tabulated in Table S1.

Table S1. Emission lifetimes (τ), radiative decay rate constant (k_{rad}), and non-radiative decay rate constant (k_{nrad}) of bare TPE-Q_4 and TPE-Q_4⊂DSP5 in water.

Compound	τ (ns)	k_{rad} (s^{-1})	k_{nrad} (s^{-1})
TPE-Q_4	1.14	5.90×10^6	8.68×10^8
TPE-Q_4⊂DSP5	5.64	9.64×10^7	8.08×10^7

5. Competition cations experiments

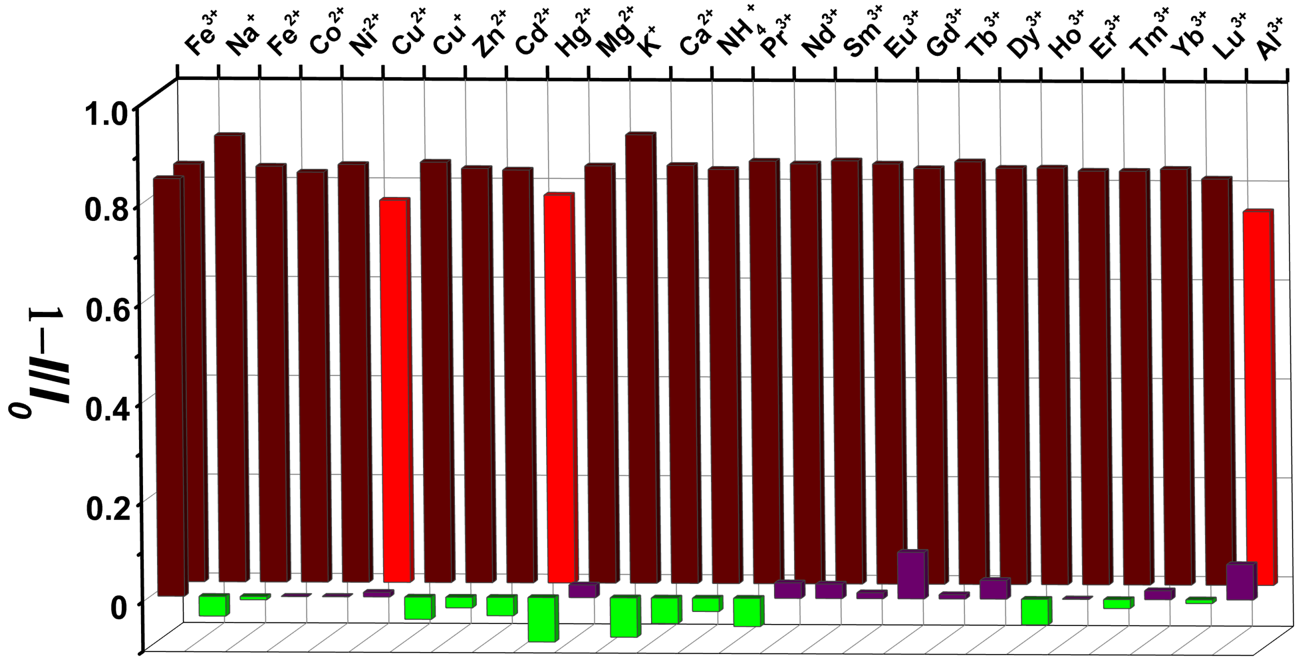

Figure S6. Fluorescence quenching degrees in competition experiments of TPE-Q_4⊂DSP5 in water. Front: TPE-Q_4⊂DSP5 with different competing cations; back: TPE-Q_4⊂DSP5 with Fe^{3+} and same equiv of cations. (Experimental conditions: λ_{ex} = 330 nm; λ_{em} = 475 nm; slit widths: Ex. 5 nm, Em. 5 nm; 298 K; [TPE-Q_4] = 1 μM, [DSP5] = 4 μM, [Fe^{3+}] = 20 μM, [Ions] = 20 μM).

6. 2D plots for selective detection of Fe^{3+}

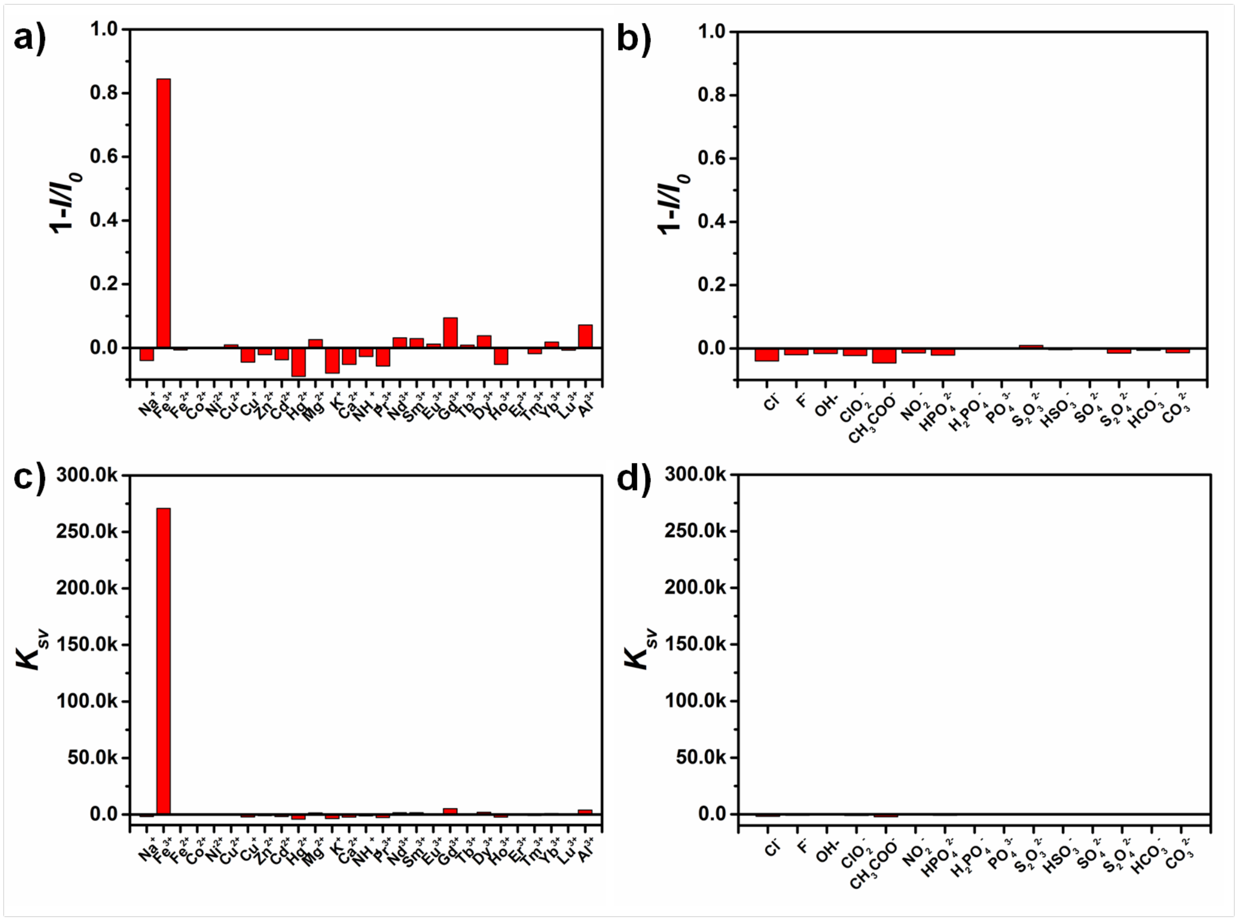

Figure S7. Fluorescence quenching degrees and the Stern–Volmer formula constants of TPE-Q_4⊂DSP5 in the presence of different cations and anions in water. 2D plots for all the M^{x+} with Cl^- (a, c) and Na^+ with N^{y-} (b, d).

7. Limit of detection

The limit of detection (LOD) was determined from the equation LOD = $K \times \delta/S$, where $K = 3$, δ is the standard deviation of the emission intensity of TPE-Q_4⊂DSP5 and S is the slope of the calibration curve of the fluorescence emission.

The result of the analysis as follow:

Linear Equation: Y = -33.4215X + 586.51671 $R^2 = 0.9966$

$S = 3.34215 \times 10^7$ $\delta = 9.57677$ (n=20) $K = 3$

LOD = $K \times \delta/S = 8.6 \times 10^{-7}$ M

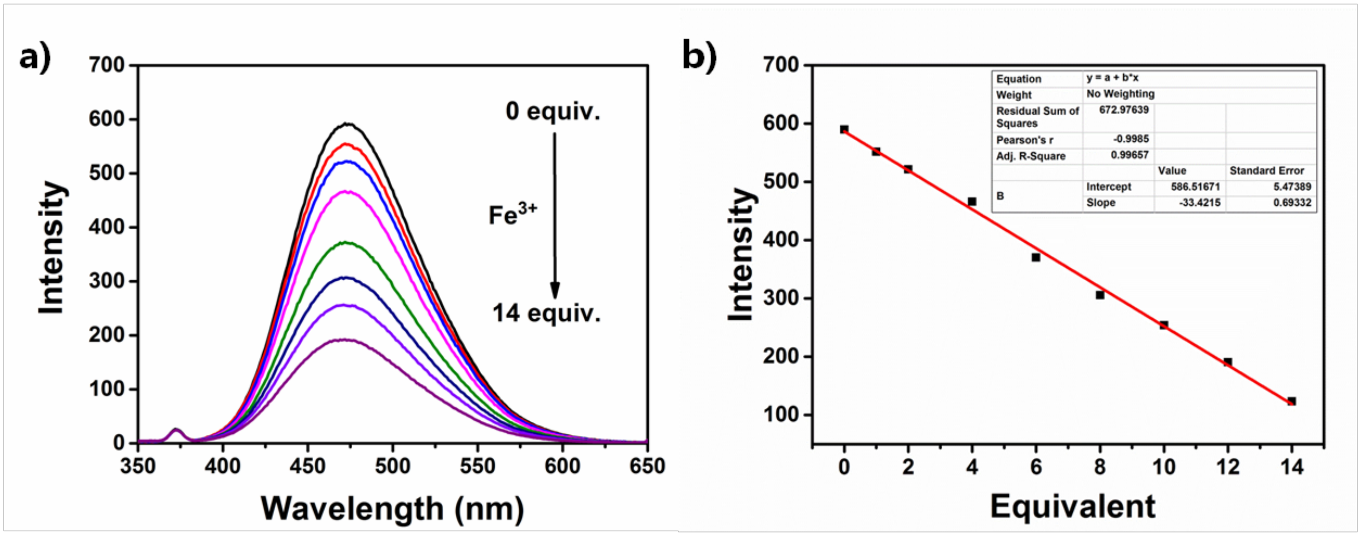

Figure S8. a) Fluorescence spectra of supramolecular assemblies TPE-Q_4⊂DSP5 aqueous solution in the presence of increasing amounts of Fe^{3+}; b) The photograph of the linear range. (Experimental conditions: λ_{ex} = 330 nm; λ_{em} = 475 nm; slit widths: Ex. 5 nm, Em. 5 nm; 298 K; [TPE-Q_4] = 1 μM, [DSP5] = 4 μM).

8. Detection of Fe^{3+} under different pH levels

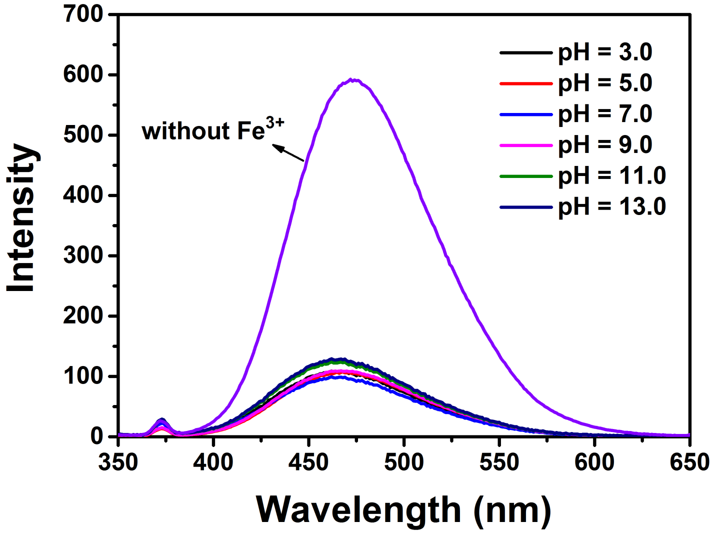

Figure S9. Fluorescence spectra of TPE-Q_4⊂DSP5 in the presence of Fe^{3+} under different pH values.

9. Size and morphology

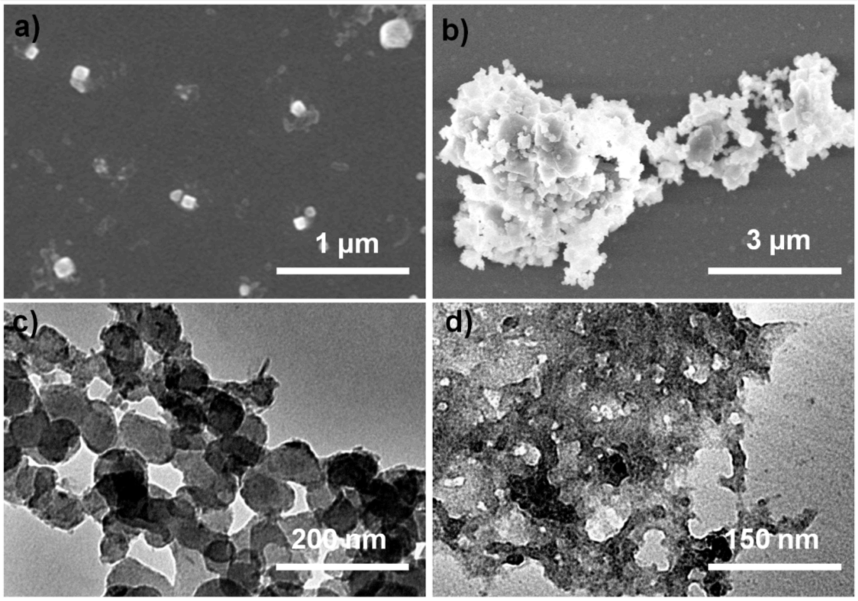

Figure S10. SEM images of a) DSP5+Fe^{3+} and b) DSNa+Fe^{3+}; TEM images of c) DSP5+Fe^{3+} and d) TPE-Q_4⊂DSP5+Fe^{3+} ([DSP5] = 10 μM, [DSNa] = 50 μM, [DSP5]/[Fe^{3+}] = 1:20, [DSNa]/[Fe^{3+}] = 1:20).

10. ^{1}H NMR experiments

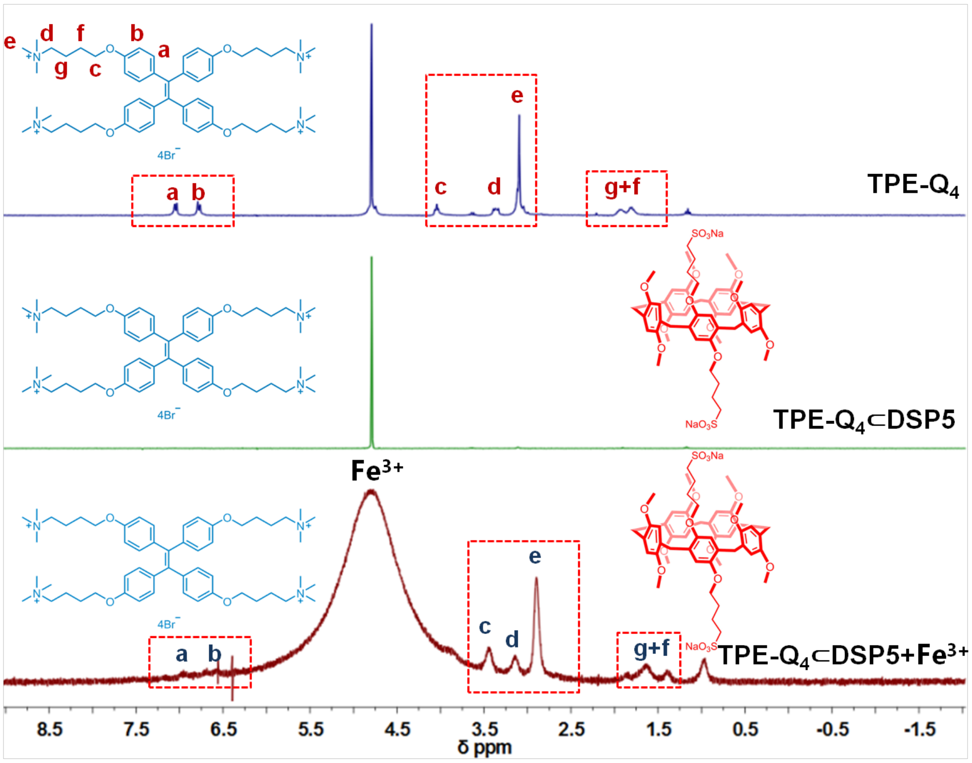

Figure S11. ^{1}H NMR spectra of TPE-Q_4, TPE-Q_4⊂DSP5 and TPE-Q_4⊂DSP5+Fe^{3+} ([TPE-Q_4] = 1.00 mM, [DSP5] = 4.00 mM, [Fe^{3+}] = 20.00 mM, 300 MHz, D_2O, 298 K).

11. Fluorescence recovery experiment

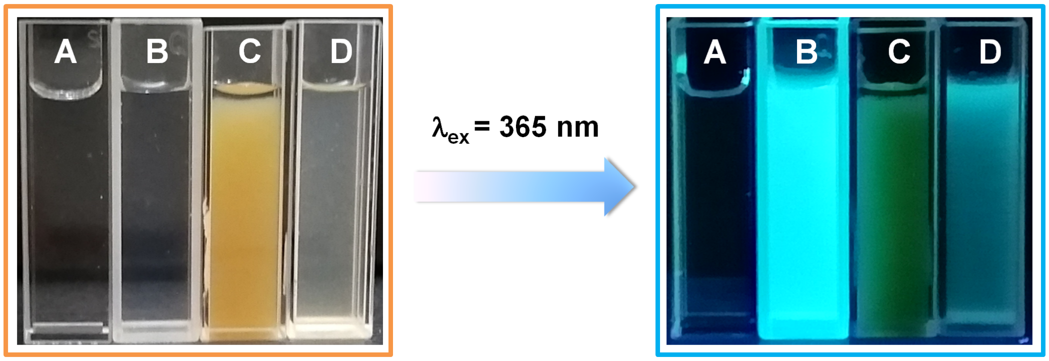

Figure S12. The pictures of (A) TPE-Q_4, (B) TPE-Q_4⊂DSP5, (C) TPE-Q_4⊂DSP5 + Fe^{3+} and (D) supernatant upon addition of DSP5 again after removing the DSP5 + Fe^{3+} precipitate.

12. Tyndall effect

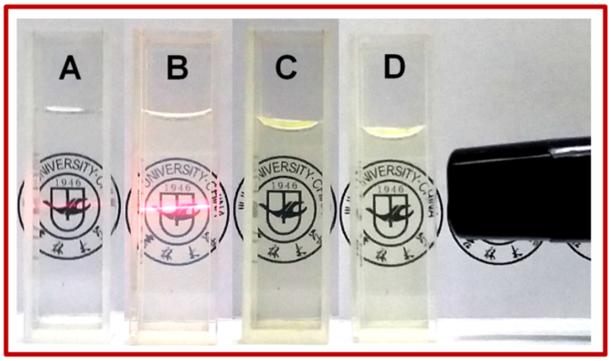

Figure S13. The pictures of (A) TPE-Q_4, (B) TPE-Q_4⊂DSP5, (C) TPE-Q_4⊂DSP5 + Fe^{3+} and (D) DSP5 + Fe^{3+} in water (after centrifugation).

13. NMR spectra

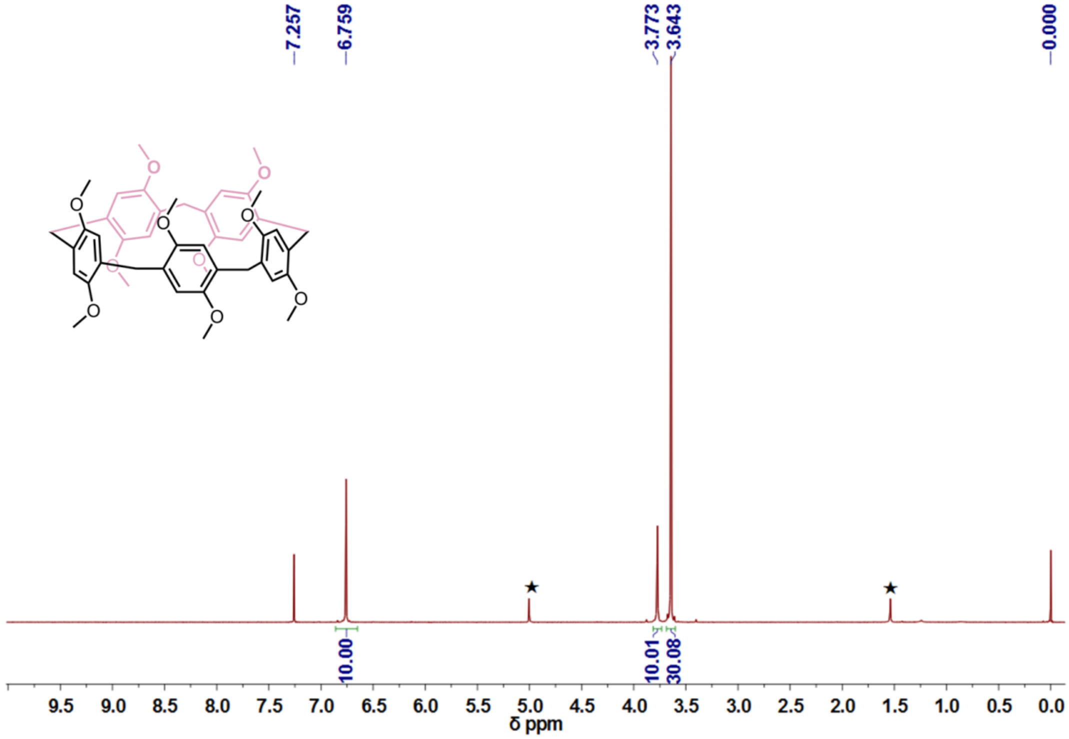

Figure S14. ^{1}H NMR spectrum (300 MHz, $CDCl_3$, 298 K) of compound **1**.

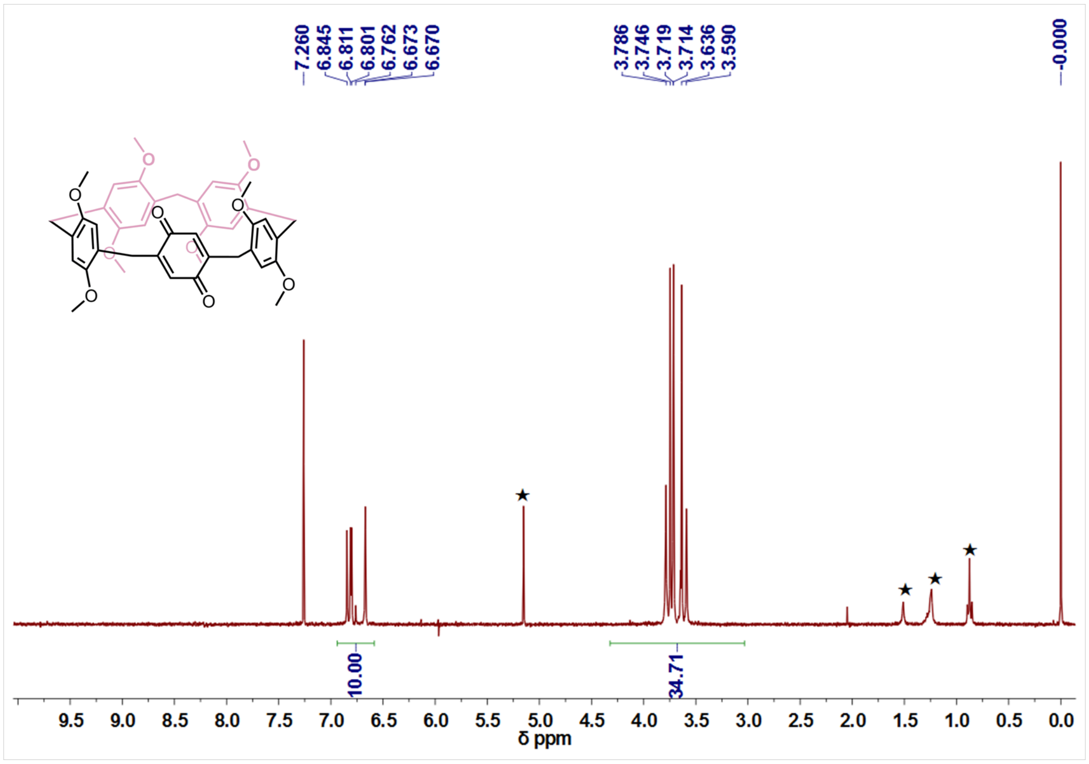

Figure S15. ^{1}H NMR spectrum (300 MHz, $CDCl_3$, 298 K) of compound **2**.

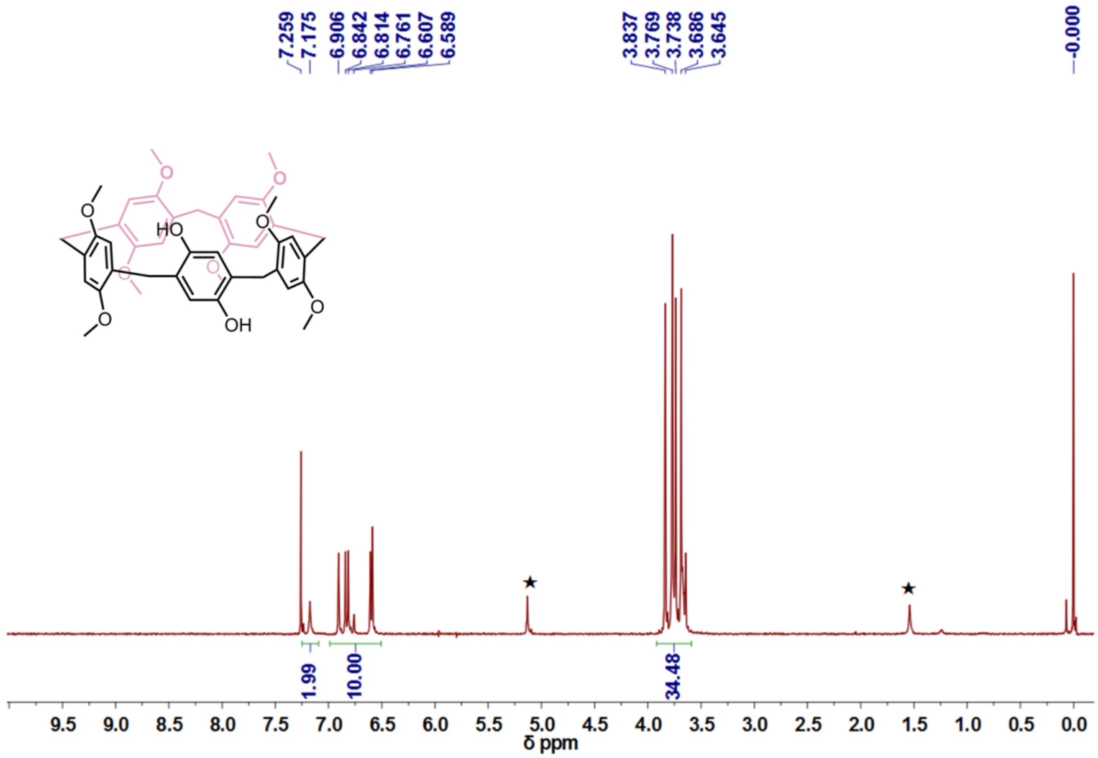

Figure S16. ^{1}H NMR spectrum (300 MHz, $CDCl_3$, 298 K) of compound **3**.

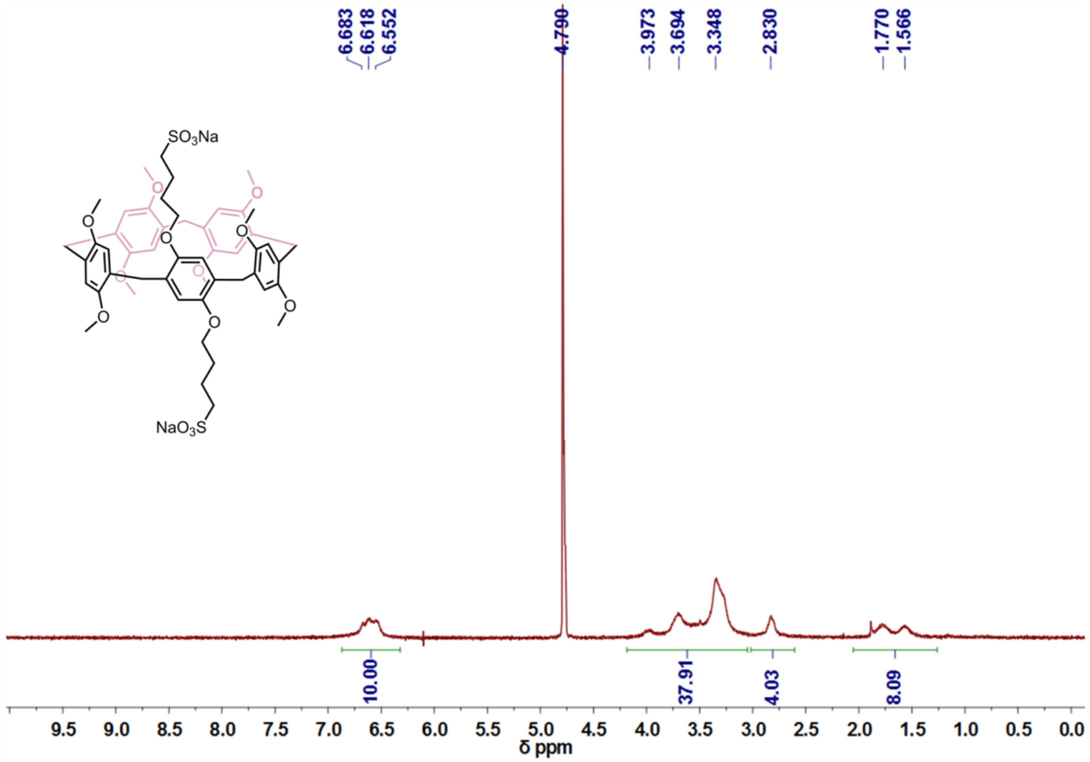

Figure S17. ^{1}H NMR spectrum (300 MHz, D_2O, 298 K) of **DSP5**.

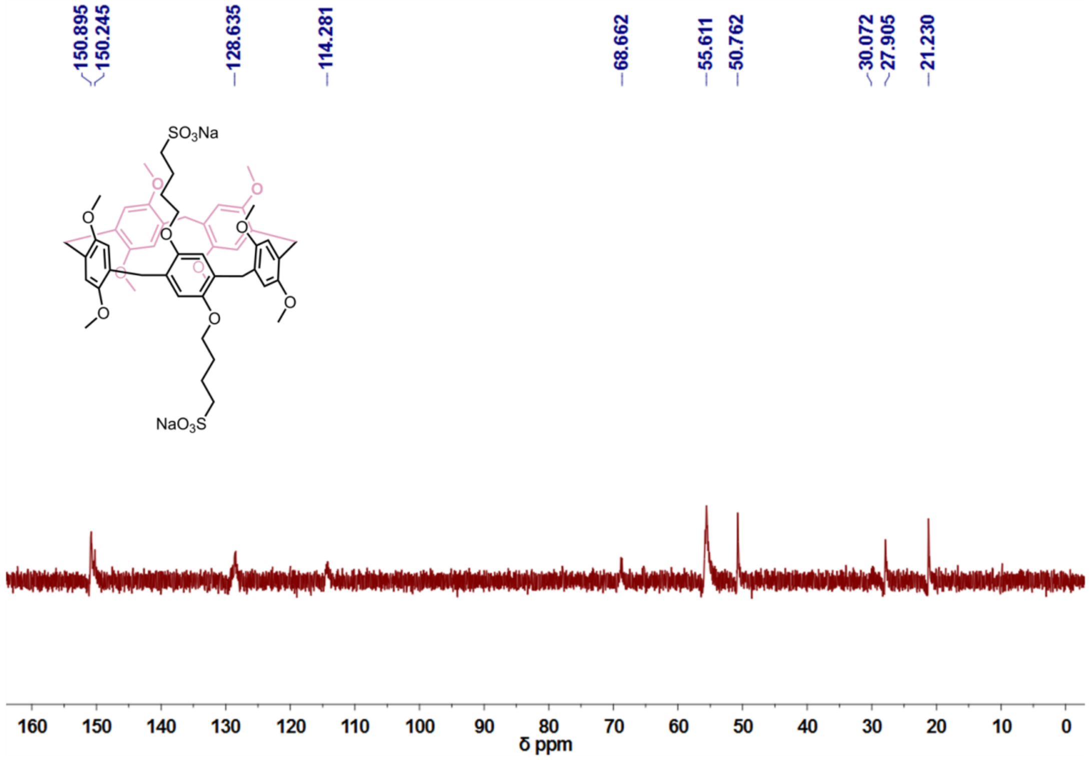

Figure S18. ^{13}C NMR spectrum (126 MHz, D_2O, 298 K) of **DSP5**.

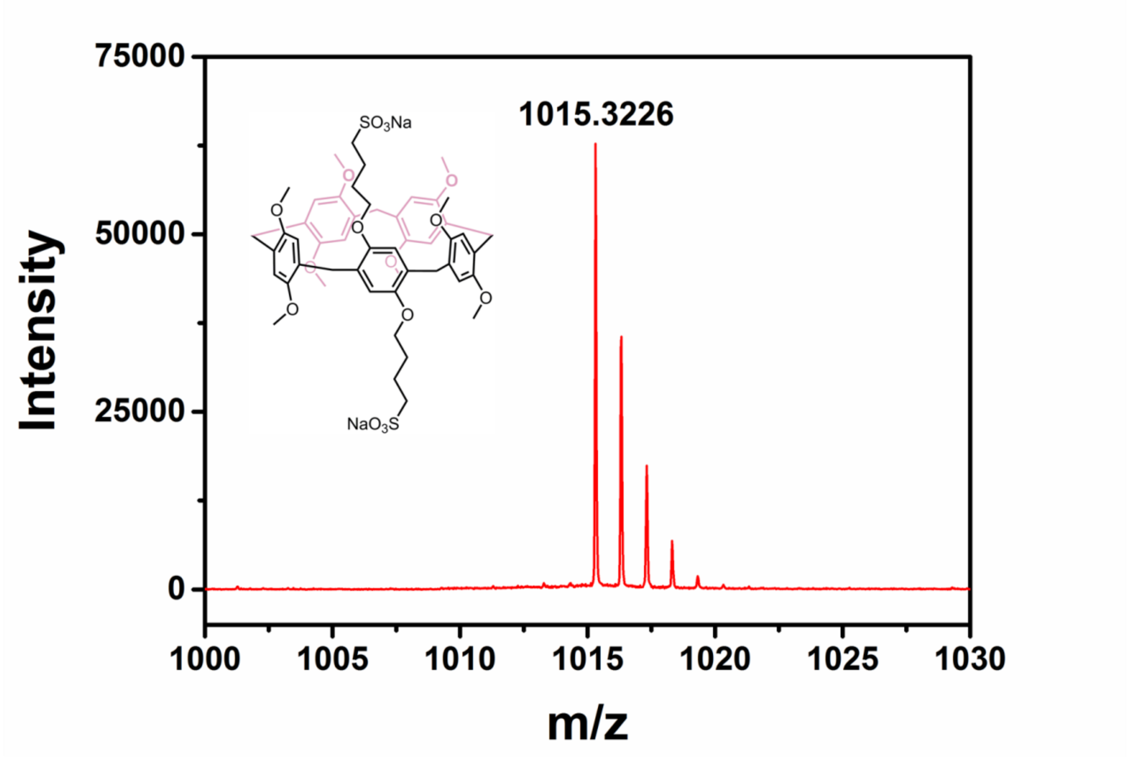

Figure S19. HRESI-MS m/z $[M-Na]^-$ calcd. for $C_{51}H_{60}O_{16}S_2Na^-$ 1015.3215, found 1015.3226.

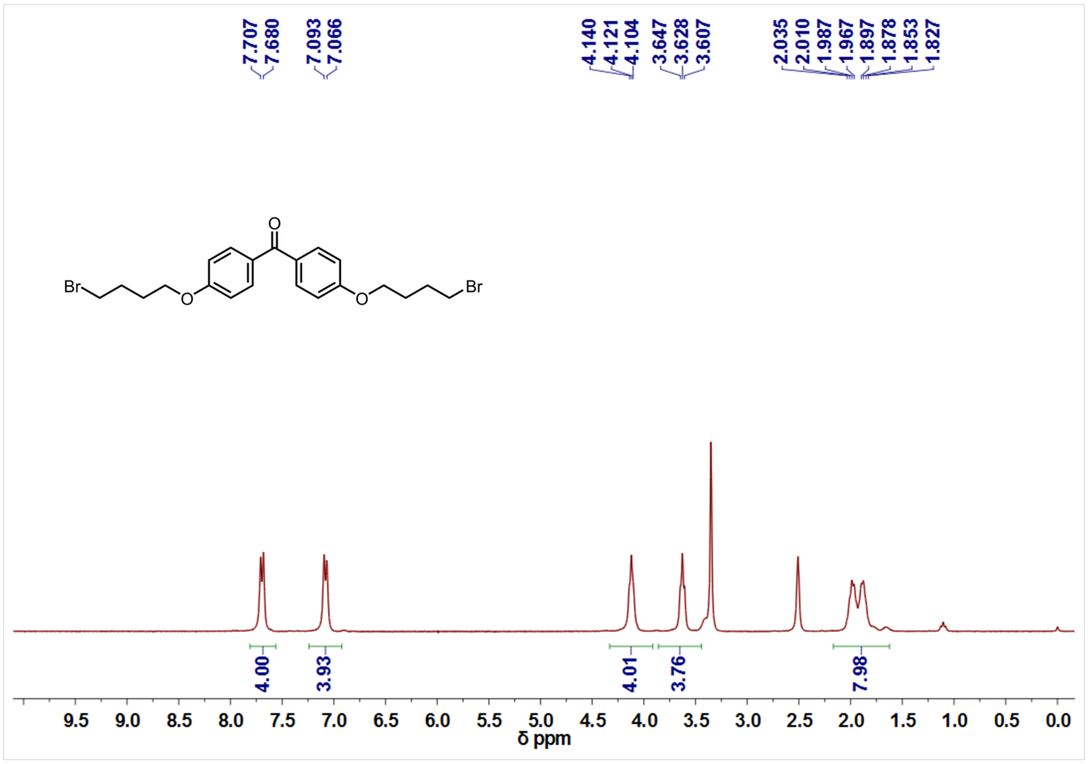

Figure S20. ^{1}H NMR spectrum (300 MHz, DMSO-d_6, 298 K) of compound **4**.

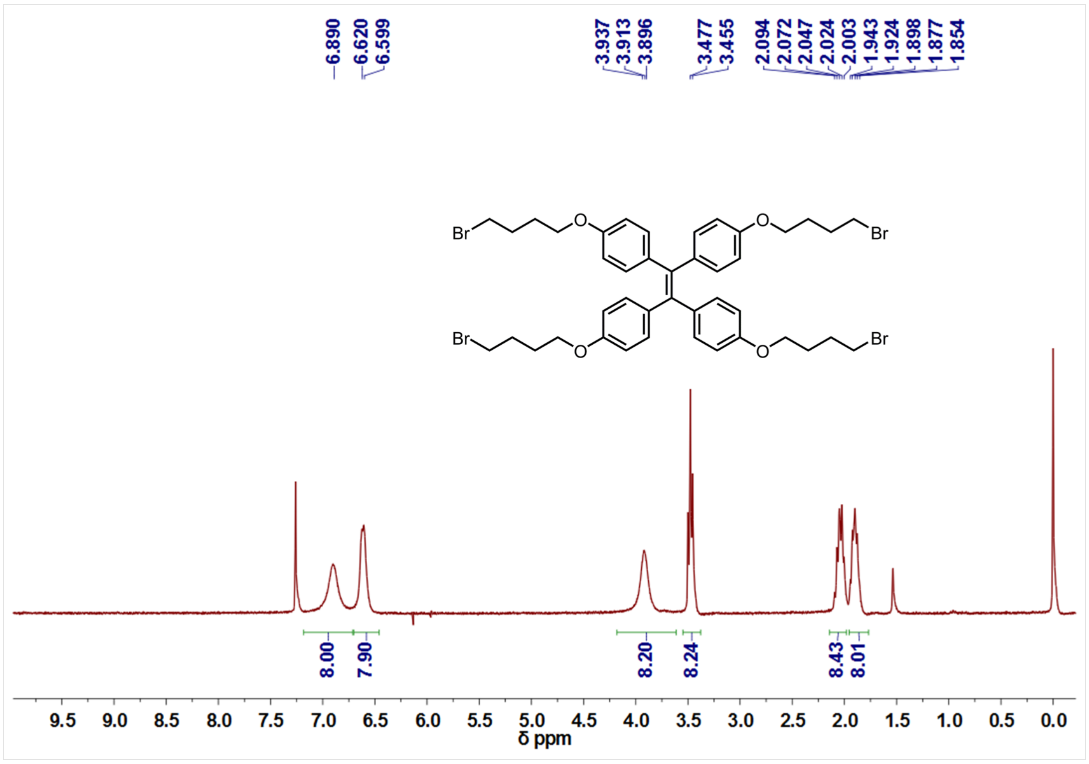

Figure S21. ^{1}H NMR spectrum (300 MHz, $CDCl_3$, 298 K) of compound **5**.

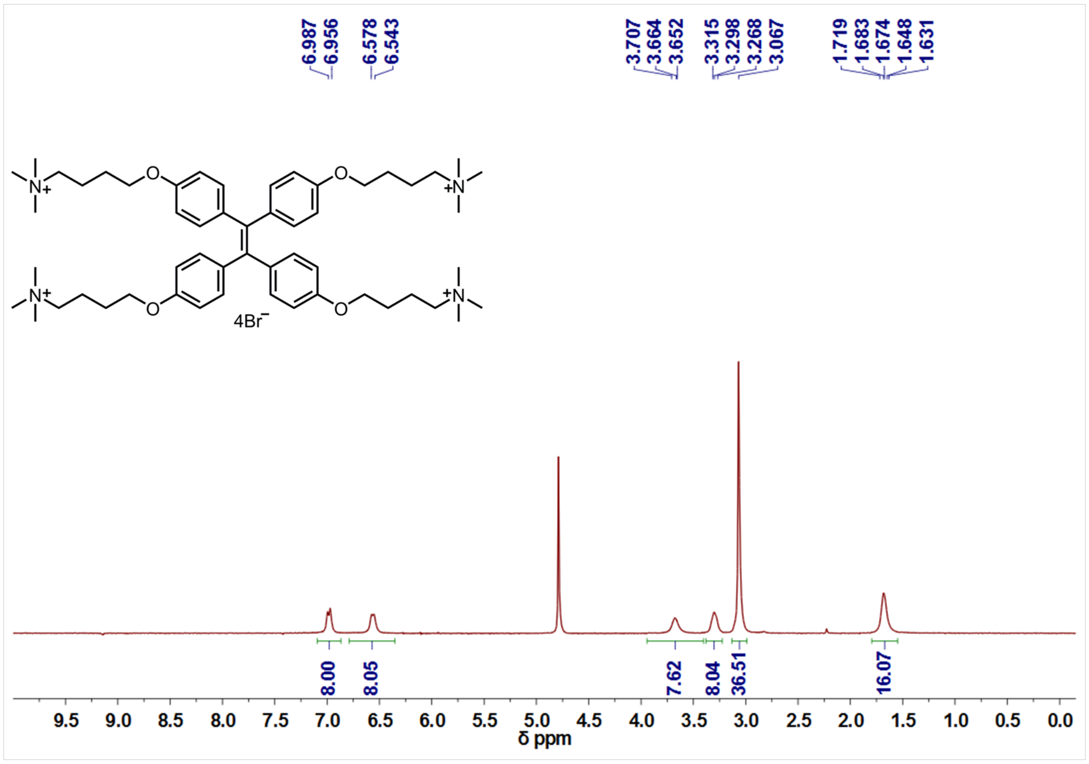

Figure S22. ^{1}H NMR spectrum (300 MHz, D_2O, 298 K) of **TPE-Q4**.

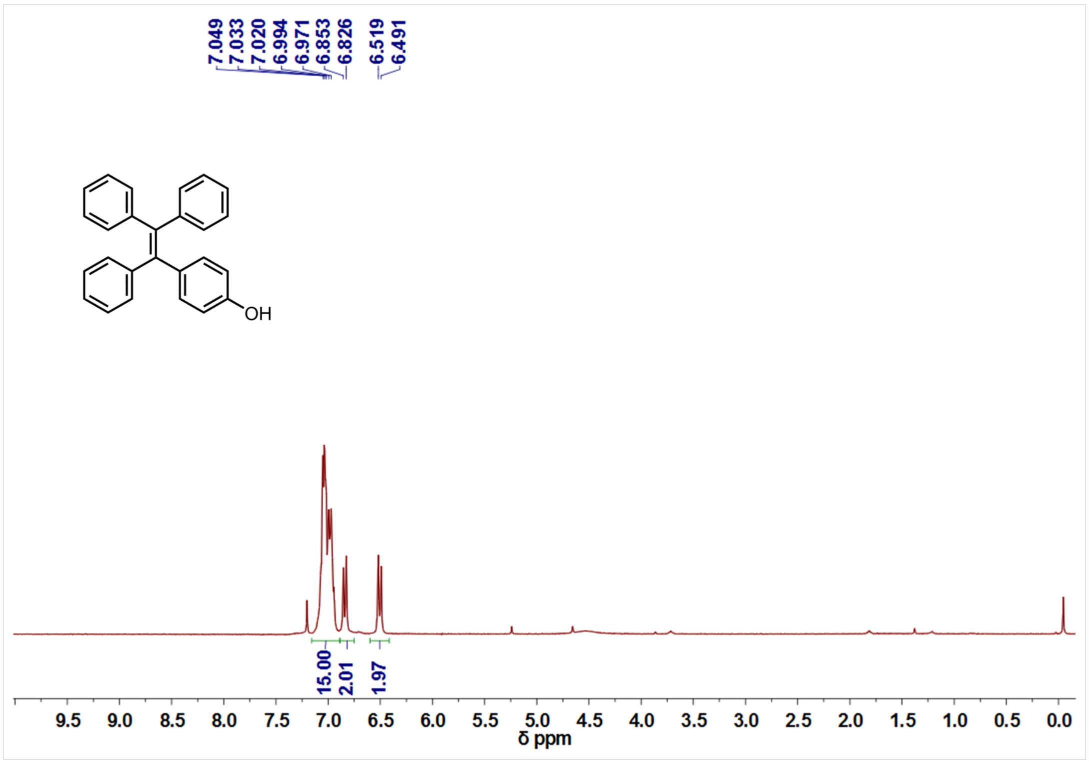

Figure S23. ^{1}H NMR spectrum (300 MHz, $CDCl_3$, 298 K) of compound **6**.

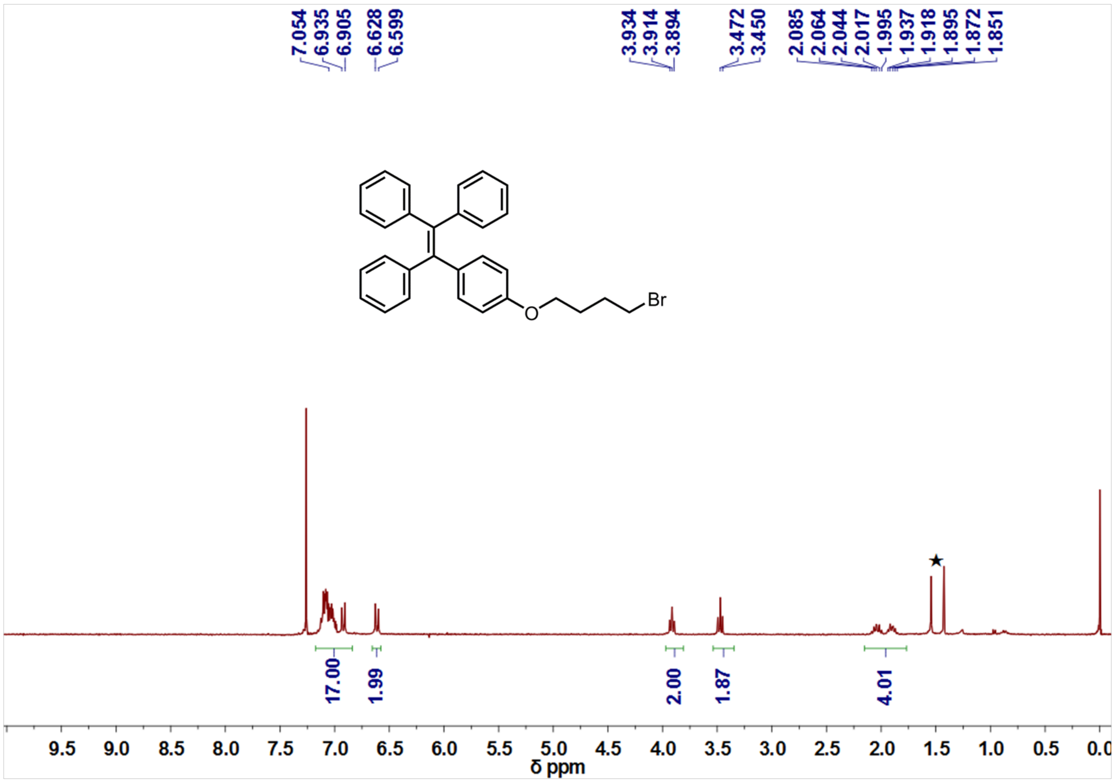

Figure S24. ^{1}H NMR spectrum (300 MHz, $CDCl_3$, 298 K) of compound **7**.

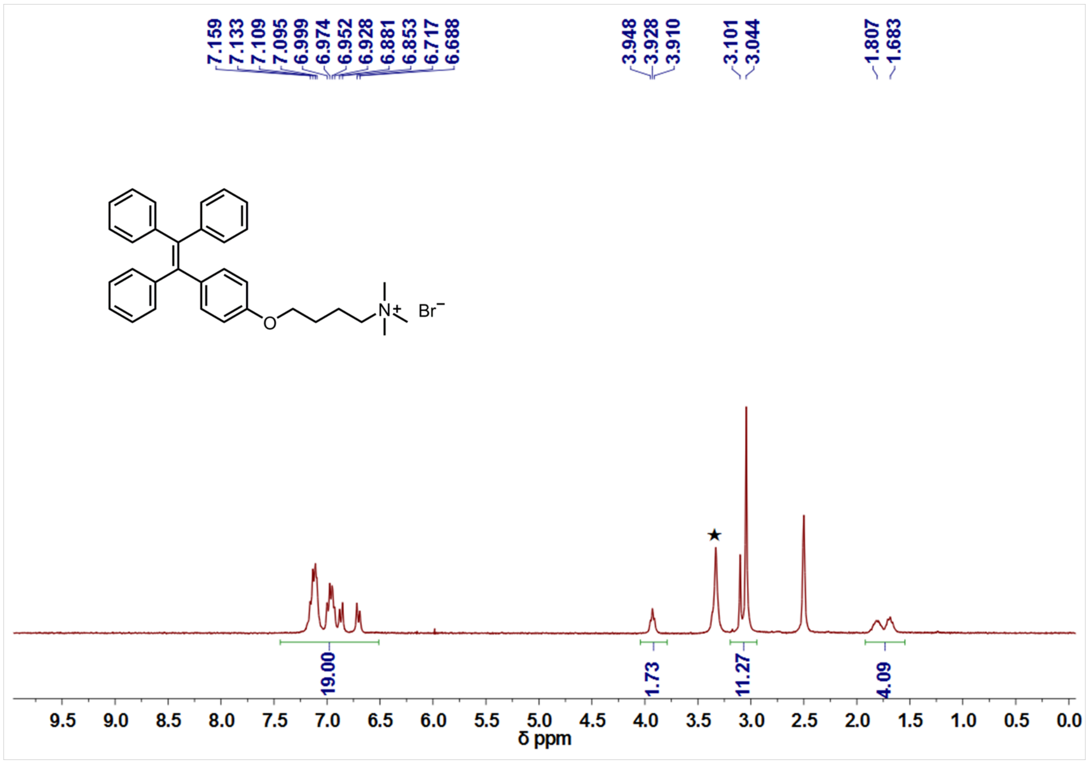

Figure S25. ^{1}H NMR spectrum (300 MHz, DMSO-d_6, 298 K) of **TPE-Q**.

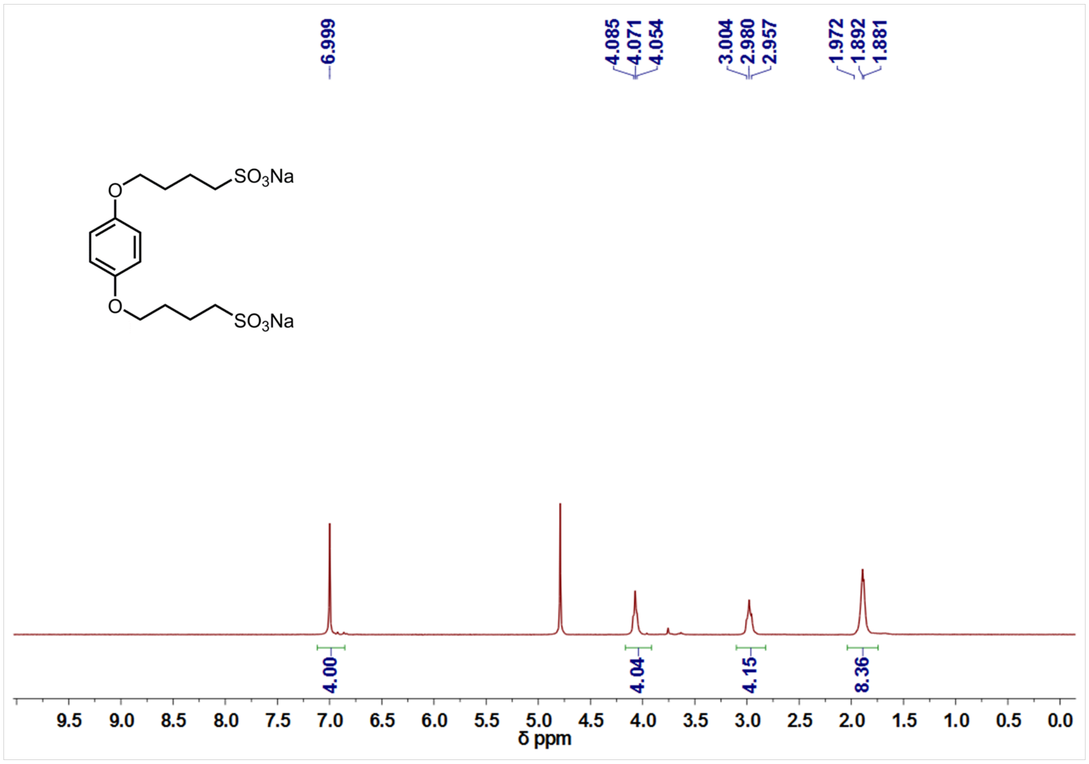

Figure S26. ^{1}H NMR spectrum (300 MHz, D_2O, 298 K) of **DSNa**.
